# Biomimetic Janus MXene membrane with bidirectional ion permselectivity for enhanced osmotic effects and iontronic logic control

**DOI:** 10.1126/sciadv.adx1184

**Published:** 2025-09-17

**Authors:** Han Qian, Hongzhao Fan, Puguang Peng, Yan Du, Xiang Li, Yanhui Liu, Feiyao Yang, Yanguang Zhou, Zhong Lin Wang, Di Wei

**Affiliations:** ^1^ Beijing Institute of Nanoenergy and Nanosystems, Chinese Academy of Sciences, Beijing 101400, People’s Republic of China.; ^2^ School of Nanoscience and Engineering, University of Chinese Academy of Sciences, Beijing 100049, People’s Republic of China.; ^3^ Department of Mechanical and Aerospace Engineering, The Hong Kong University of Science and Technology, Clear Water Bay, Kowloon, Hong Kong SAR, People’s Republic of China.; ^4^ Centre for Photonic Devices and Sensors, University of Cambridge, 9 JJ Thomson Avenue, Cambridge, CB3 0FA, UK.

## Abstract

Osmotic efficiency is fundamentally governed by the balance between membrane ion selectivity and permeability, a challenge central to both biological signal transmission and sustainable energy conversion. Conventional membranes are constrained to unidirectional transport of either cations or anions, severely limiting their versatility and performance. Inspired by the chloride voltage-gated channel 5 (ClC-5), we engineered a biomimetic Janus NP-MXene membrane featuring subnanochannels (~6.0 angstrom) and exceptional structural integrity, enabling controlled, simultaneous Na^+^/Cl^−^ transport with unprecedented permselectivity. Under a 50-fold salinity gradient, the NP-MXene membrane achieved a record power density of 85.1 watts per square meter and an osmotic potential of 181.5 millivolts, the highest reported for a single device. Harnessing ion-specific signals from multi-ion transport, we further demonstrated an iontronic transistor capable of modulating ion flow by salinity gradients, eliminating the need for external gate voltage. This advance enables encoded signals and robotic control for advanced human-machine interfaces. The scalable fabrication of nanofluidic channels facilitates high-performance iontronics for efficient energy-information flow.

## Teaser

A Janus membrane enables bidirectional ion permselectivity for energy-information flow in nanoconfined iontronics.

## INTRODUCTION

Osmotic effects are fundamental in biological systems, not only for neural information transmission but also for sustainable energy conversion (*1*, *2*). In biological cellular membranes, channel proteins permit certain types of ion flow across the membrane driven by osmotic effects from salinity gradient, responsible for nervous impulses, muscle contractions, and physiological sensing. Specifically, in the nervous system, osmotic effects of ions such as sodium (Na^+^), potassium (K^+^), and calcium (Ca^2+^) are essential for the generation of action potentials, which are the basis of nerve impulses (fig. S1A) (*3*, *4*). The precise control of ion flow across neuron membranes, facilitated by ion channels and driven by salinity gradients, allows for the rapid and precious signal transmission. The periodic closing and opening of Na^+^/K^+^ channels achieve the action potential generation and ensure the continuous transmission of electrical signals, while Ca^2+^ dynamics at synapses also play a crucial role in neurotransmitter release and synaptic plasticity, vital for learning and memory (*5*). In addition to roles in information transmission, the osmotic effects also play a pivotal role in adenosine triphosphate (ATP) generation in biological systems (fig. S1B) (*6*, *7*). The proton gradient in mitochondria, created by the transport of H^+^ ions across the inner membrane during respiration, drives ATP synthesis via ATP synthase. This chemiosmotic coupling mechanism converts osmotic energy into chemical energy, powering various cellular life activities (*8*, *9*). Similarly, in photosynthetic organisms, osmotic effects across thylakoid membranes generate the proton motive force necessary for converting light energy into chemical energy (*10*). Therefore, osmotic effects serve the dual role in biological systems, underpinning both the efficient energy flow and the precious information flow (*11*). We propose that efficient energy flow and precise information transmission are intrinsically unified as an “energy-information flow,” a fundamental principle underlying diverse biological processes and offering critical insights into the design of artificial ion channels and ion transport mechanisms (*12*).

**Fig. 1.**
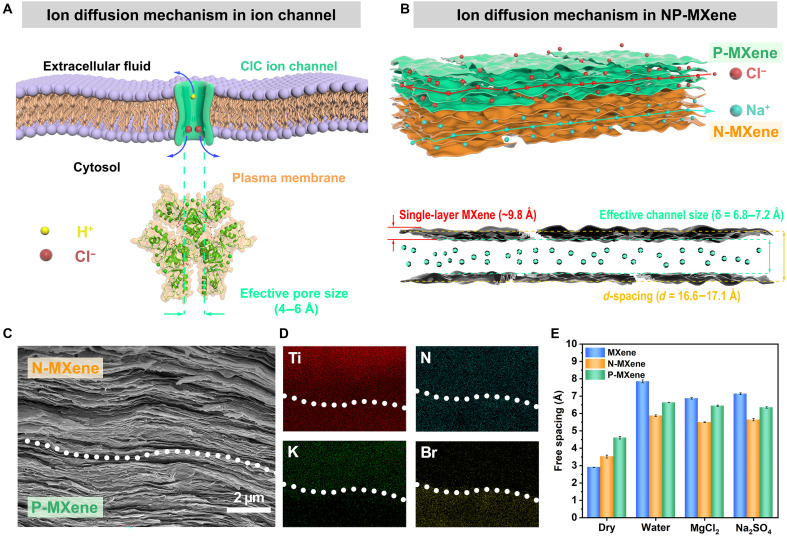
Schematics of the biological ion channel and biomimetic NP-MXene nanoconfined channels. (**A**) Schematic of ion diffusion mechanism of ClC-5 with effective pore size. (**B**) Schematic of ion diffusion mechanism in biomimetic NP-MXene channels with subnanochannel size. (**C**) Cross-sectional SEM image of the NP-MXene. A typical lamellar structure can be found in the NP-MXene and exhibits no obvious boundary between the N-MXene and P-MXene layers. (**D**) Energy-dispersive x-ray spectroscopy (EDS) elemental mapping of Ti, N, K, and Br in the cross section of the NP-MXene after soaking in KBr solution (0.1 M). (**E**) The free spacings of MXene, N-MXene, and P-MXene membranes obtained from the x-ray diffraction (XRD) patterns.

Diverging from the macroscopic ion transport behavior, nanochannels reveal a host of unconventional phenomena, including overlapping electrical double layers (*5*, *13*, *14*), ionic coulomb blockade (*15*, *16*), and superionic states (*17*, *18*). The discovery of these phenomena clearly suggests that nanoconfined conditions provide a unique approach to manipulating solvated ion transport for both fundamental understanding and application exploration (*19*–*25*). Building on this, the studies of artificial ion channels mimicking their biological counterparts could deepen our understanding of complex ion transport in biological systems and advance the study of ion dynamics in nanoconfined conditions (*4*, *5*, *26*–*30*). For instance, Xiong *et al.* (*31*) introduced a polyelectrolyte-confined fluidic memristor capable of executing various neuromorphic functions, effectively mimicking both electric pulse patterns and chemical-electrical signal transduction. Moreover, Li *et al.* (*32*) developed an ultraselective artificial sodium channel that not only emulates the functionality of biological ion channels but also offers notable potential for applications in enhanced osmotic effects and future neuromorphic information processing. However, these advancements depend critically on the in situ growth of active materials within nanopipettes, a process that is both intricate and challenging to scale up. In addition, since conventional membranes are constrained to unidirectional transport of either cations or anions, the maximum membrane voltage under a 50-fold salinity gradient was limited to ~100 mV (*t_+_* or *t_−_* = 1), which remarkably restricted the practical application of osmotic effects. Here, inspired by the chloride voltage-gated channel 5 (ClC-5), which mediates Cl^−^ influx coupled with H^+^ efflux and displays pronounced outward rectifying currents (Fig. 1A) (*33*, *34*), we report a facile solution–based chemical modification strategy to fabricate a biomimetic Janus MXene membrane featuring negatively and positively charged domains (NP-MXene) and subnanochannels (Fig. 1B). The NP-MXene is composed of EDTA-modified cation-selective channel (N-MXene) and polydiallyl dimethyl ammonium (PDDA)–modified anion-selective channel (P-MXene). This approach, involving the restacking of exfoliated two-dimensional (2D) nanofluidic sheets, enables the creation of tunable and stable laminar conduction pathways while ensuring compatibility with scalable fabrication methods such as printing for large-scale production (*13*, *33*). Under a 50-fold salinity gradient, the biomimetic NP-MXene achieved an unprecedented membrane voltage of 181.5 mV, surpassing the theoretical limit of ~100 mV of conventional single cation-/anion-selective membranes, and achieved an ultrahigh osmotic power density of 81.5 W m^−2^. Beyond power generation, these ion channels also enable information transmission, where the salinity gradient plays a central role in energy-based information flow, enabling encoded signals and robotic control for advanced human-machine interfaces. This study offers valuable insights into the design and development of artificial ion channel membranes, enabling the regulation of multi-ion transport in enhanced osmotic effects and presenting a precious approach to advancing iontronic logic control and neuromorphic information processing through biomimetic ion channels with nanoconfined effects.

## RESULTS

### Fabrication and characterization of NP-MXene

To fabricate the NP-MXene permselective membrane with subnanochannels, the Al layer was first etched from the parent Ti_3_AlC_2_ phase to obtain single- or few-layer Ti_3_C_2_T*_x_* MXene nanosheets (*34*, *35*). These nanosheets were then modified with EDTA and PDDA (figs. S2 and S3), yielding two types of Ti_3_C_2_T*_x_* nanosheets (N-MXene and P-MXene) with opposite charge polarities, enabling uniform and stable dispersion in aqueous solution (figs. S4 and S5). The as-synthesized nanosheets processed a thickness of ~1.5 nm (fig. S6A), close to the theoretical thickness of Ti_3_C_2_T*_x_* MXene nanosheets (1.0 nm) (*36*). The uniform distribution of nitrogen elements and the increased thickness of the modified N-MXene and P-MXene nanosheets confirm the successful modification with EDTA and PDDA (figs. S6 to S10). When examined under transmission electron microscopy (TEM), these nanosheets appeared ultrathin and flattened, and the selected area electron diffraction mode revealed a single-crystal diffraction pattern, further confirming their monolayer features (figs. S11 to S13). Because of the existence of oxygen-containing functional groups (─OH and ─O) and Lewis acid Ti sites on the surface of MXene nanosheets (fig. S14), the Ti_3_C_2_T*_x_* MXene nanosheets exhibited a negative charge in aqueous solution, with a zeta potential of −35.49 mV (fig. S15). Therefore, during the modification of the nanosheets with EDTA, these surface-active sites enable EDTA to bond with MXene nanosheets in multiple forms through both covalent bonding [with Lewis acid Ti (*35*, *37*)] and noncovalent bonding [with hydrogen bond (*38*)] after simple mixing, resulting in N-MXene nanosheets. Similarly, P-MXene is obtained by adjusting the surface charge through the formation of hydrogen bonds between the polyelectrolyte PDDA and the Ti_3_C_2_T*_x_* MXene surface via electrostatic attraction (*39*). As shown in fig. S15, N-MXene exhibited an increased zeta potential (−47.91 mV) compared to the original MXene, while P-MXene showed a reversal in zeta potential (+46.41 mV). The emerging of the N 1s spectra in the x-ray photoelectron spectroscopy (XPS) of N-MXene and P-MXene, compared to MXene, also implied the successful grafting of EDTA and PDDA onto the nanosheets (figs. S16 and S17).

By sequentially filtering N-MXene and P-MXene solutions onto porous cellulose acetate filter paper, a flexible self-supporting NP-MXene Janus membrane was obtained (fig. S18). The NP-MXene membrane exhibited different colors on its two sides, with the N-MXene side showing the original purple-black color of MXene and the P-MXene side appearing black. Both sides showed a uniform, defect-free surface with good hydrophilicity (figs. S19 and S20). Cross-sectional images from scanning electron microscopy (SEM) images revealed the typical layered structure of the NP-MXene membrane. After soaking in KBr solution (0.1 M), the corresponding energy-dispersive x-ray spectroscopy (EDS) elemental mapping presented characteristic signals of K and Br elements, indicating the multi-ion selectivity of NP-MXene channels. The N-MXene and P-MXene layers were well-connected without noticeable gaps (Fig. 1, C and D). On the N-MXene side, EDTA is cross-linked with MXene nanosheets through Ti-COO^−^ covalent bonds and hydrogen bonds, while PDDA on the P-MXene side is firmly attached to MXene nanosheets via electrostatic attraction and hydrogen bonds, as revealed by XPS (figs. S21 and S22) and Fourier transform infrared (FTIR) spectroscopy (fig. S23). In addition, N-MXene and P-MXene membranes with similar thicknesses to the NP-MXene membrane were obtained using the same vacuum-assisted filtration method. The thickness of all three membranes was measured to be ~5 μm using a 3D profilometer (figs. S24 to S26). Compared to the original MXene membrane, the N-MXene and P-MXene membranes exhibited larger interlayer spacing (*d*-spacing) under dry conditions, indicating successful modification of the MXene nanosheets (Fig. 1E and fig. S27). The interlayer spacing measured by high-resolution transmission electron microscopy images was consistent with XRD data, demonstrating channel alignment and spatial uniformity (fig. S28). In the aqueous environments, the interlayer spacing of all three membranes increased, with the unmodified MXene membrane expanding by 170%. However, because of hydrogen and covalent bonding interaction, the N-MXene and P-MXene membranes presented reduced swelling, maintaining stable free spacing of channels between 5 and 7 Å (fig. S29). Even in saline solutions, the free spacing of channels still remained consistent, which is fundamental for biomimetic ion channel design (Fig. 1E and fig. S27).

### Ion selectivity and permeability investigation

**Fig. 2.**
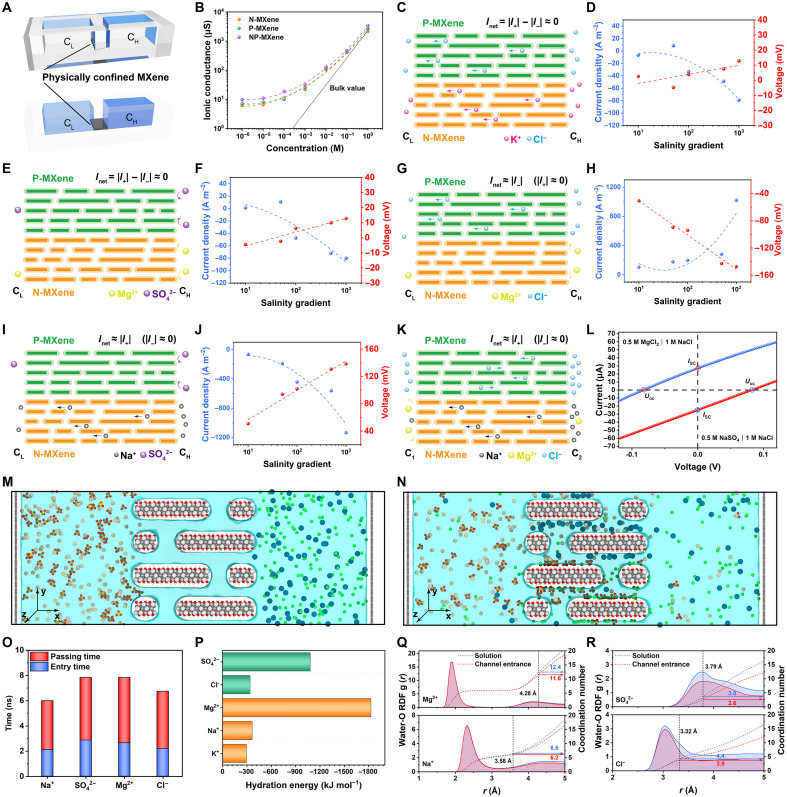
Ion transport performance of biomimetic NP-MXene nanoconfined channels. (**A**) Schematic of electrochemical testing device. The NP-MXene membrane with nanoconfined channels connects two ionic reservoirs with low concentration (*C*_L_) and high concentration (*C*_H_). (**B**) Transmembrane ionic conductance of the N-MXene, P-MXene, and NP-MXene membranes. (**C**) Schematic of the ion diffusion in NP-MXene with KCl salinity gradients. (**D**) The osmotic potential (*V*_OS_) and osmotic current density (*J*) of the NP-MXene with KCl salinity gradients. (**E**) Schematic of NP-MXene with MgSO_4_ salinity gradients. (**F**) *V*_OS_ and *J* of the NP-MXene with MgSO_4_ salinity gradients. (**G**) Schematic of NP-MXene with MgCl_2_ salinity gradients. (**H**) *V*_OS_ and *J* of the NP-MXene with MgCl_2_ salinity gradients. (**I**) Schematic of NP-MXene with Na_2_SO_4_ salinity gradients. (**J**) *V*_OS_ and *J* of the NP-MXene with Na_2_SO_4_ salinity gradients. (**K**) Schematic of NP-MXene with the left side (*C*_1_) being a high concentration of MgCl_2_ and a low concentration of NaCl solution and the right side (*C*_2_) being a high concentration of NaCl and a low concentration of MgCl_2_ solution. (**L**) The *IV* curves of two salinity gradient systems [system 1: (*C*_1_) 0.5 M MgCl_2_ and 0.02 M NaCl | (*C*_2_) 1 M NaCl and 0.01 M MgCl_2_; system 2: (*C*_1_) 0.5 M Na_2_SO_4_ and 0.02 M NaCl | (*C*_2_) 1 M NaCl and 0.01 M Na_2_SO_4_]. (**M**) The initial state and (**N**) final state of the NP-MXene channels model in Na_2_SO_4_ | MgCl_2_ salinity gradient system. (**O**) Average entry time and passing time of ions passing the NP-MXene channels in Na_2_SO_4_ | MgCl_2_ salinity gradient system. (**P**) The hydration energy of Na^+^, Mg^2+^, Cl^−^, and SO_4_^2−^ (table S1). (**Q** and **R**) Radial distribution function (RDF) of oxygen in water molecules around Na^+^, Mg^2+^, Cl^−^, and SO_4_^2−^ locating at the solution and NP-MXene subnanochannel entrance (table S2).

As a self-supporting membrane, NP-MXene channels can be directly pressed into an acrylic device and sealed with acrylic tape (*34*). The membrane was positioned between two reservoirs filled with different salt solutions, creating a physically confined MXene osmotic power source (Fig. 2A and fig. S30). This confined permselective membrane could maintain a more stable ion channel size under aqueous conditions (*40*). The ion transport property of NP-MXene channels was explored based on a KCl system due to the similar bulk ion mobility and hydration radii of K^+^ and Cl^−^ (*41*). A pair of Ag/AgCl standard electrodes with agar-saturated KCl salt bridges were used to eliminate the influence of redox reactions (*42*, *43*). Applying the structure of above device with KCl solutions of the same concentration on both sides, the corresponding transmembrane ionic conductance exhibited typical surface charge–governed ion transport (*44*, *45*). In detail, when the KCl concentration exceeded 10^−3^ M, it showed a linear relationship similar to that of the bulk system. However, at lower concentrations, the conductance gradually deviated from the bulk solution value, forming a plateau (Fig. 2B).

The ion selectivity of the NP-MXene channels was subsequently examined by imposing a chemical potential gradient within a KCl salinity gradient system (Fig. 2C) (*41*, *46*). In this system, the salinity gradient ratio of the salt solutions on both sides was defined as *C*_1_/*C*_2_, where *C*_1_ and *C*_2_ represent the salt concentrations in the left and right reservoirs, respectively. For single salt solutions, this ratio is denoted as *C*_H_/*C*_L_, with *C*_H_ and *C*_L_ indicating the high concentration on the right and the low concentration on the left. Here, *C*_L_ was fixed at 1 mM, while *C*_H_ was varied from 10, 50, 100, 500, to 1000 mM, corresponding to salinity gradients of 10-, 50-, 100-, 500-, and 1000-fold, respectively. Figure S31 shows the *IV* curves of the salinity gradient system, illustrating the osmotic potential (*V*_OS_) at zero current (open-circuit voltage) and the osmotic current (*I*_SC_) at zero voltage (short-circuit current), both generated by the ion selective permeability of the membrane (*34*). Unlike typical salinity gradient systems, the NP-MXene channels are divided into cation-selective N-MXene and anion-selective P-MXene, allowing it to be selective for both cations and anions simultaneously (Fig. 1B). Therefore, in the salinity gradient system shown in Fig. 2C, the high concentrations of K^+^ and Cl^−^ on the right side *C*_H_ will diffuse to the left side *C*_L_. Because of the similar ion mobility and hydration radius of K^+^ and Cl^−^, this results in a near-zero osmotic current and osmotic potential (Fig. 2D).

Because of the size effect (*47*–*49*) and differences in adsorption energy (*41*), the subnanochannels of NP-MXene posed higher transporting resistance to divalent ions such as Mg^2+^ and SO_4_^2−^, demonstrating selectivity between divalent and monovalent ions. In a custom-made H-shaped device, the left side (*C*_1_) contained mixed solution of 0.5 M MgCl_2_ and 0.5 M Na_2_SO_4_, while the right side (*C*_2_) contained deionized water (fig. S32). The NP-MXene channels demonstrated high monovalent/divalent selectivity for Na^+^/Mg^2+^ and Cl^−^/SO_4_^2−^, with selectivity ratios of 110.34 and 91.45, respectively (figs. S33 and S34). The underlying mechanisms will be analyzed in detail in the following section. Consequently, in the MgSO_4_ salinity gradient system, limited cation and anion diffusion leads to a near-zero osmotic current and osmotic potential (Fig. 2, E and F, and fig. S35). By adjusting the two salinity gradient systems so that one ion type could efficiently transport while the other is not, it is possible to achieve electrochemical performance similar to that of traditional single cation-/anion-selective membranes (Fig. 2, G and H). As shown in Fig. 2 (I and J), osmotic potential is approximately proportional to the salinity gradient (red curve), whereas the absolute value of osmotic current increases exponentially (blue curve), which could be attributed to the unipolar transport of Cl^−^ and Na^+^ in the two systems (figs. S36 and S37).

If the two reservoirs contain different salt solutions, where the left side (*C*_1_) has a mixture of 0.5 M MgCl_2_ and 0.02 M NaCl and the right side (*C*_2_) has a mixture of 1 M NaCl and 0.01 M MgCl_2_, the solutions still form a 50-fold salinity gradient (Fig. 2K). Since the Cl^−^ concentration is identical on both sides, there is no ion diffusion driven by a chemical potential gradient. Because of the high transporting resistance to Mg^2+^, the primary ion flow in this system is the diffusion of Na^+^ from the high concentration on the right (*C*_2_) to the low concentration on the left (*C*_1_), resulting in a *V*_OS_ of 82.3 mV and an *I*_SC_ of 27.3 μA (Fig. 2L). Similarly, in the (*C*_1_) 0.5 M Na_2_SO_4_ and 0.02 M NaCl | (*C*_2_) 1 M NaCl and 0.01 M Na_2_SO_4_ system, the primary ion flow is Cl^−^, achieving a *V*_OS_ of 82.4 mV and an *I_SC_* of 24.9 μA (Fig. 2L and fig. S38).

### Modeling of ion diffusion in NP-MXene

To further investigate the ion transport characteristics of NP-MXene channels, molecular dynamics simulations were used to study the transport behavior of different ions within the channels (*47*, *50*, *51*). The NP-MXene model, similar to the experimental results, was constructed with free spacing of 6.5 Å for both N-MXene and P-MXene channels (fig. S39). To quantify the differences in ion transport dynamics, the process was divided into two key steps: ion entry into the channel and ion passage through the channel. First, for both the KCl salinity gradient system and the Na_2_SO_4_ salinity gradient system, cations and anions orderly passed through their respective channels. Specifically, K^+^ and Mg^2+^ passed through the negatively charged N-MXene channels, while Cl^−^ and SO_4_^2−^ passed through the positively charged P-MXene channels (figs. S40 and S41). Moreover, a comparison of the ion quantities transported between monovalent and divalent ions revealed that the number of monovalent ions (K^+^ and Cl^−^) transported over the same period was remarkably higher than that of divalent ions (Mg^2+^ and SO_4_^2−^). The quantity of monovalent ions increased with time, whereas the number of divalent ions remained very low, almost zero, growing at a very slow rate over time (figs. S42 and S43). A further comparison of the time taken for ions to enter and pass through the channel in both systems showed that the entry and passing times for K^+^ and Cl^−^ were similar and shorter than those for Mg^2+^ and SO_4_^2−^, which is consistent with the experimental results (fig. S44).

In the Na_2_SO_4_ | MgCl_2_ salinity gradient system, the ordered transport of cations and anions, as well as the differences in transport dynamics between monovalent and divalent ions, was also observed (Fig. 2, M and N, and fig. S45). Specifically, in this system, Na_2_SO_4_ solution was placed on the left side and MgCl_2_ solution on the right, and after diffusion, the selectivity of the NP-MXene channels for K^+^/Mg^2+^ and Cl^−^/SO_4_^2−^ was found to be 4 and 8, respectively (fig. S46). Notably, because of computational limitations, ion selectivity was underestimated relative to experimental findings (*34*, *35*). Compared with the single-salinity gradient system described earlier, both ion entry and transit times exhibited a slight increase. Nevertheless, monovalent ions still demonstrated shorter entry and transit times than divalent ions, resulting in a reduced overall transmembrane transport time for monovalent ions (Fig. 2O). Ion entry into channels with subnanometer dimensions, smaller than the ions’ hydrated diameters, induces partial dehydration, facilitating a more conforming fit within the confined space (*17*, *50*, *52*, *53*). The energy required for ion dehydration is positively correlated with their respective full hydration energies (*47*, *48*, *54*). Since the hydration energies of cations follow the order K^+^ (−295 kJ mol^−1^) < Na^+^ (−365 kJ mol^−1^) « Mg^2+^ (−1830 kJ mol^−1^), and the hydration energies of anions follow Cl^−^ (−340 kJ mol^−1^) « SO_4_^2−^ (−1080 kJ mol^−1^), it indicates that the dehydration energy barriers at the channel entry should follow a similar sequence for different ions (Fig. 2P) (*35*, *55*). This is consistent with the experimentally observed ion permeation rates, where Na^+^ > Mg^2+^ and Cl^−^ > SO_4_^2−^ (fig. S33). Moreover, the radial distribution function analysis shows a notable difference in the degree of dehydration between Na^+^ and Mg^2+^, with each ion losing 0.8 (from 12.4 to 11.6) and 0.3 (from 6.5 to 6.2) water molecules from their hydration shells, respectively (Fig. 2Q). A similar phenomenon is observed in the dehydration process of Cl^−^ and SO_4_^2−^ (Fig. 2R).

As ions traverse the channel, partial dehydration within the subnanochannels introduces resistance, with the extent of resistance varying among ions. 2D potential energy diagrams of ion transport reveal that the channel interior exhibits high energy barrier, hindering ion passage. Furthermore, the energy barrier for divalent ions is higher than that for monovalent ions, amplifying the selective transport effect (fig. S47), indicating that monovalent ions (K^+^ and Cl^−^) encounter less resistance during transport compared to divalent ions (Mg^2+^ and SO_4_^2−^). In addition, EDTA and PDDA within the channels not only modulate channel dimensions and surface charge but also play a critical role in ion transport and separation (*35*, *41*, *56*). The deprotonated carboxyl groups (─COO^−^) of EDTA, together with two electronegative N atoms, can act as ligands that coordinate with alkaline earth metals and transition metal ions, forming more chemically stable complexes (*35*). This remarkably hinders the transport of Mg^2+^ but does not affect K^+^ in the same way. Meanwhile, because of electrostatic attraction being proportional to charge magnitude, EDTA and PDDA attract divalent ions more strongly than monovalent ions under the same conditions. It is worth noting that, during horizontal ion migration within the NP-MXene channel, a minor fraction of ions undergoes cross-channel (vertical) transport. However, the predominant pathway remains horizontal migration (figs. S48 and S49). Furthermore, by analyzing the steady-state spatial distributions of Na^+^ and Cl^−^ near the channel, we observe that ion concentrations at the entrances of both N-MXene and P-MXene layers in the NP-MXene membrane are higher compared to their respective pure membranes (N-MXene or P-MXene membranes), while the concentrations at the outlet slightly decreased (fig. S50). This enhancement at the entry region could be attributed to the spatially asymmetric charge distribution in the Janus heterostructure, which dynamically regulates local ion distribution. Last, density functional theory (DFT) calculations were conducted to study the adsorption energies (*E*_ads_) of EDTA-cation and PDDA-anion combinations. The DFT results showed that the *E*_ads_ between EDTA and Mg^2+^ (−5.91 eV) is much higher than that with monovalent Na^+^ (−1.58 eV) or K^+^ (−1.22 eV). Similarly, the *E*_ads_ between PDDA and SO_4_^2−^ (−4.64 eV) is higher than that with monovalent Cl^−^ (−1.09 eV) (fig. S51). In summary, divalent ions face a greater transport energy barrier while passing through the channel (*35*).

### Enhanced osmotic effects with scalable implementation

**Fig. 3.**
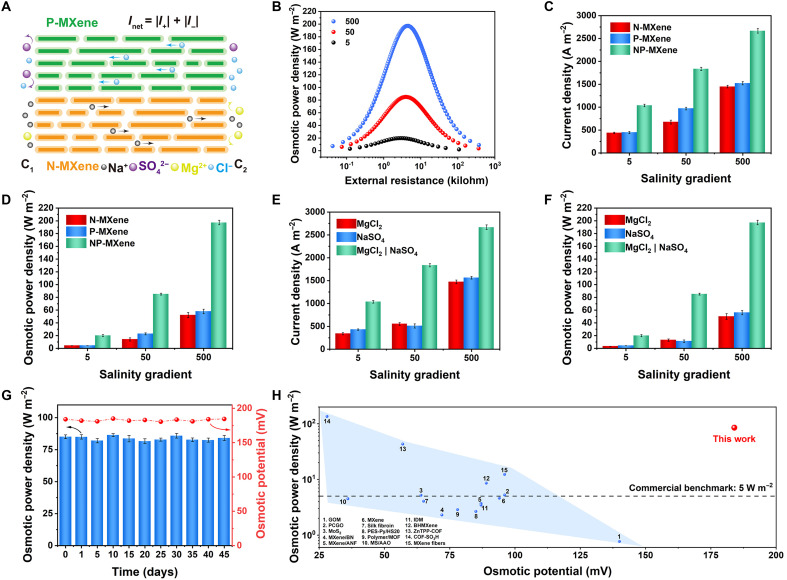
The enhanced osmotic effects of biomimetic NP-MXene membrane with nanoconfined ion channels in Na_2_SO_4_ | MgCl_2_ salinity gradient system. (**A**) Schematic of the ion diffusion in NP-MXene with the left side (*C*_1_) being a high concentration of MgCl_2_ and a low concentration of Na_2_SO_4_ solution and the right side (*C*_2_) being a high concentration of Na_2_SO_4_ and a low concentration of MgCl_2_ solution. (**B**) The areal osmotic power density of a NP-MXene as a function of the increasing external resistance under three salinity gradients in Na_2_SO_4_ | MgCl_2_ salinity gradient system. (**C**) Comparison of osmotic current densities for N-MXene, P-MXene, and NP-MXene under three salinity gradients in the Na_2_SO_4_ | MgCl_2_ salinity gradient system. (**D**) Comparison of areal osmotic power densities for N-MXene, P-MXene, and NP-MXene under three salinity gradients in the Na_2_SO_4_ | MgCl_2_ salinity gradient system. (**E**) Comparison of osmotic current densities for NP-MXene under three salinity gradients in the MgCl_2_, Na_2_SO_4_, and Na_2_SO_4_ | MgCl_2_ salinity gradient systems. (**F**) Comparison of areal osmotic power densities for NP-MXene under three salinity gradients in the MgCl_2_, Na_2_SO_4_, and Na_2_SO_4_ | MgCl_2_ salinity gradient systems. (**G**) The long-term stability of the NP-MXene osmotic power source. The membrane was immersed in the testing solutions all the time, and before each measurement, the testing solutions were replenished. (**H**) Comparison of the NP-MXene areal osmotic power source with other reported osmotic power sources for the osmotic power density and osmotic potential. GOM, graphene oxide membrane; PCGO, neuromorphic graphene oxide; PES, pyridine pendants; MOF, metal organic framework; MS/AAO, mesoporous silica/macroporous aluminaI; DM, ionic diode membrane.

After investigating the ion selectivity and permeability performance of the NP-MXene channels, we constructed a Na_2_SO_4_ | MgCl_2_ salinity gradient system to evaluate its performance on enhanced osmotic effects. In such osmotic power source, the left side (*C*_1_) contained a mixture of 0.5 M Na_2_SO_4_ and 0.01 M MgCl_2_, while the right side (*C*_2_) contained 0.5 M MgCl_2_ and 0.01 M Na_2_SO_4_, forming a 50-fold salinity gradient (Fig. 3A). Figure S52 shows the typical *IV* curve of the NP-MXene channels under a 50-fold Na_2_SO_4_ | MgCl_2_ salinity gradient system, generating a remarkably high *V*_OS_ of 181.5 mV and an *I*_SC_ of 45.9 μA, corresponding to a current density of up to 1836.7 A m^−2^ (fig. S53). By constructing a bidirectional ion transport mechanism, Na^+^ and Cl^−^ achieve simultaneous transport, resulting in the effective synergistic of the osmotic potential and current. This is the key to the NP-MXene channels’ outstanding performance. The areal osmotic power density could be calculated using ***P*** = ***I***^2^***R***, where ***I*** is the current and ***R*** is the external load resistance (*41*). At an intermediate load resistance of 3.9 kilohm, the maximum power density reached 85.1 W m^−2^ (Fig. 3B). Ultimately, the NP-MXene osmotic power source achieved an ultrahigh power density of 197.3 W m^−2^ under a 500-fold salinity gradient (Fig. 3B).

To further investigate the ion transport performance of the NP-MXene channels in the Na_2_SO_4_ | MgCl_2_ salinity gradient system, we replaced the NP-MXene with N-MXene and P-MXene channels of the same size and tested their respective *IV* curves (figs. S54 and S55). According to the calculations, under a 50-fold salinity gradient, the cation transference number (*t_+_*) of the N-MXene and the anion transference number (*t_−_*) of the P-MXene were 0.95 and 0.91, respectively, with corresponding current densities of 673.7 and 970.1 A m^−2^ (Fig. 3C). The power densities of the N-MXene and P-MXene osmotic power sources under a 50-fold salinity gradient were 14.0 and 22.7 W m^−2^, respectively, far lower than that of NP-MXene under the same conditions (Fig. 3D). This difference primarily arises from NP-MXene’s capacity to synergistically transport both cations and anions, thereby modulating co-ion distribution outside the channel and alleviating steric hindrance for counter-ion transport (*17*, *41*, *57*). This synergistic transport effect becomes more pronounced under a 500-fold salinity gradient. Because of ion concentration polarization, the N-MXene and P-MXene osmotic power sources exhibit power densities of 52.1 and 57.8 W m^−2^, respectively, while the NP-MXene osmotic power source still delivers a high power density of 197.3 W m^−2^. In addition, the ion transport performance of NP-MXene channels was also explored in the MgCl_2_ | MgCl_2_ and Na_2_SO_4_ | Na_2_SO_4_ salinity gradient systems. In the former, the main ion flow consisted of Cl^−^ passing through the P-MXene, generating a *V*_OS_ of 92.4 mV and an osmotic power density of 13.0 W m^−2^. Similarly, Na^+^ ions passing through the N-MXene produced a *V*_OS_ of 84.8 mV and an osmotic power density of 11.2 W m^−2^, both remarkably lower than the NP-MXene osmotic power source under equivalent conditions (Fig. 3, E and F, and figs. S56 and S57), suggesting that the biomimetic NP-MXene channels synergistically enhanced the osmotic effects by efficient anion-cation regulation. In addition, we examined the influence of structural parameters of the NP-MXene channels. Varying the ratio of N-MXene to P-MXene layers had negligible effects on ion selectivity and osmotic performance (fig. S58). We further performed scaling experiments by independently varying the channel length (*L*) and width (*W*) (fig. S59). While the osmotic potential showed only minor dependence on these dimensions, the osmotic current scaled approximately in accordance with Ohm’s law (figs. S60 and S61). These results suggest that channel length and width primarily affect transmembrane resistance, without altering the charge-governed ion selectivity or energy conversion efficiency (*34*, *58*).

To evaluate the operational stability of the NP-MXene channels’ osmotic effects, a 45-day osmotic power density test was conducted. The results showed that the initial maximum osmotic power density was 85.1 W m^−2^ under a 50-fold Na_2_SO_4_ | MgCl_2_ salinity gradient, and even after 45 days, there was no notable decay, indicating excellent long-term operational stability (Fig. 3G). Meanwhile, Fig. 3H summarizes our osmotic power generation performance in comparison to the latest reported results in terms of osmotic potential and power density. Notably, the NP-MXene channels achieved unprecedented osmotic potential and ultrahigh osmotic power density (table S3). Meanwhile, MXene stands out as one of the most pH-resilient 2D nanofluidic materials (*34*). Building on its intrinsic chemical stability, the NP-MXene membrane further demonstrated exceptional pH tolerance, likely attributable to the synergistic effect of its uniquely bidirectional ion transport pathways (figs. S62 and S63). Compared to the original MXene membrane, the NP-MXene membrane maintained robust mechanical integrity after 1 week of bending and soaking in a mixed Na_2_SO_4_/MgCl_2_ solution, with no signs of separation. This stability might be attributed to the enhanced interfacial adhesion arising from coulombic attraction between the oppositely charged N-MXene and P-MXene layers, conferring excellent antiswelling properties and long-term structural durability suitable for practical applications (figs. S64 and S65). To further demonstrate the scalability of NP-MXene channels, the 2D nanofluidic channels were constructed by extrusion printing of N-MXene and P-MXene aqueous inks (figs. S66 to S68). These channels achieved a high osmotic power density of 40.32 W m^−2^ under a 50-fold Na_2_SO_4_ | MgCl_2_ salinity gradient, three times higher than current benchmarks, maintaining enhanced osmotic effects over large areas, the area was an order of magnitude higher than conventional large-area devices for osmotic power generation (table S4) (*41*). This is primarily due to the bidirectional synergistic transport of cations and anions, which simultaneously enhance both ion selectivity and permeability, enhancing the NP-MXene channels’ osmotic effects.

### Iontronic logic control

**Fig. 4.**
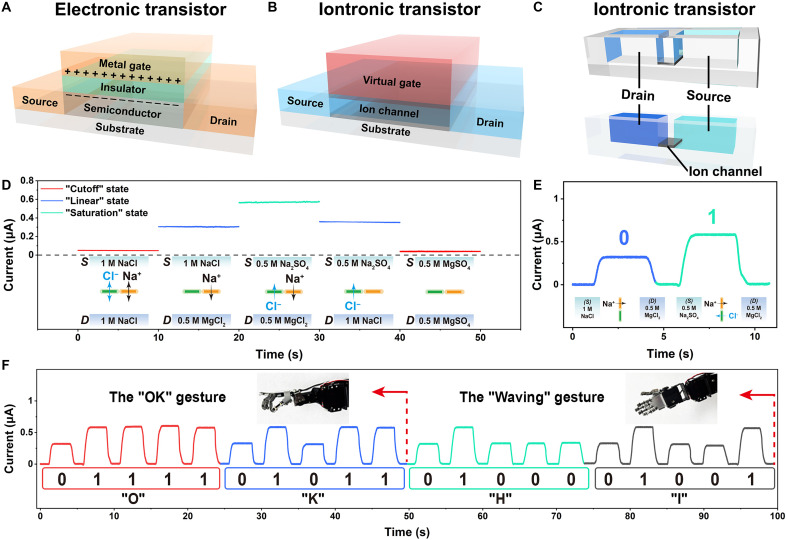
Iontronic logic control of NP-MXene iontronic transistor. (**A**) The electronic transistor with electrons as charge carriers. (**B**) The iontronic transistor with ions as charge carriers has virtual gates. (**C**) Schematic of the NP-MXene iontronic transistor with source, drain, and ion channel. (**D**) Current signal switches when a stimulus is applied (ion). (**E**) Define low and high current signal levels (0 and 1), achieved through single-ion (Na^+^) and dual-ion (Na^+^ and Cl^−^) transport, respectively. (**F**) Information transmission in human-machine interaction based on biomimetic ion flow of NP-MXene iontronic transistor.

Beyond enhanced osmotic effects, the multi-ion transport properties of NP-MXene can facilitate information transmission by harnessing salinity gradients as an energy source for information flow. In biological systems, the voltage-gated ion channel (Fig. 1A) (*59*) functions similarly, i.e., changes in the cell’s transmembrane potential drive ion flow through the channel, powered by salinity gradients to transmit information. The channel’s functional units, dominated by charged particles on its molecular subunits, respond sensitively to minor membrane voltage changes, triggering gate opening or closing (*59*, *60*). Thus, the voltage-gated channel serves as life’s transistor, enabling information transmission and logic control (*60*). Now, the electronic transistor, an essential three-terminal device comprising a source, drain, and gate, stands as one of the most important inventions of the last century for information transmission and logic control. By integrating numerous complementary semiconductor transistors into compact chips, this technology facilitates high-speed data processing and complex computations, laying the groundwork for the information age (*60*). Figure 4A illustrates the architecture of a typical top-gated electronic transistor, where semiconductor materials function as channels for electron or hole transport between the source and drain, separated by an insulating layer (*61*). However, although various devices are constantly being developed, the Moore’s law in silicone-based electronic transistor is approaching its limits, and the energy efficiency of the most sophisticated electronic transistor to mimic the iontronic logic circuit in single-cell organisms is still inferior to their natural counterpart (*4*, *13*, *62*). Even today, the most advanced silicon-based electronics struggle to replicate brain functions, far from achieving the efficiency of the human brain, which operates on just 12 W of energy (*63*). Consequently, it will be difficult to achieve advanced information processing functions in an energy efficient way, such as highly efficient pattern recognition, intelligent reasoning, embedded sensing and computing, and brain-machine interfaces (*30*, *64*).

To address this, we select ions, natural carriers of most biological activities, as charge carriers. Their varied valencies, sizes, polarizabilities, and other characteristics enable a low-energy, high-efficiency iontronic transistor suitable for diverse information transmission applications. A simple iontronic transistor was developed on the basis of NP-MXene channels. Free from external gate voltage control, the device enables regulated ion transport and distinct signal output via ion signals generated by a virtual gate formed through the salinity gradient between the source and drain (Fig. 4, B and C). Conventional electronic transistors typically operate in three states (fig. S69): “cutoff” state, “linear” state, and “saturation” state, where current will change with voltage. For our NP-MXene iontronic transistors, changes in external gate voltage are replaced by salinity gradient. When different concentrations of salt solutions are used on either side, the relationship between the salinity gradient and output current density can be obtained, which resembles the characteristic curve of electronic transistors (fig. S70). Such iontronic transistor similarly exhibits a cutoff state (KCl and MgSO_4_), a linear state (MgCl_2_ and Na_2_SO_4_), and a saturation state (MgCl_2_ | Na_2_SO_4_). It is worth noting that, in this section, we used a simplified configuration rather than the complex mixed solution system with well-defined salinity gradients. Even when a pure single-salt solution is used as the ion reservoir on one side, the system still enables effective iontronic logic control and precise information flow. Furthermore, this system enables rapid transitions by different ion signals. When the solution in the ion reservoir changes, the transported ions change accordingly, and the current signal changes rapidly in response to the different states of the iontronic transistor (Fig. 4D and fig. S71). This feature enables control of various robotic movements through distinct current threshold settings. To advance toward autonomous iontronic control, we used a fully enclosed, sealed fluidic system integrated with programmable syringe or peristaltic pumps for automated solution exchange on both sides of the NP-MXene membrane (figs. S72 to S74). Specifically, the source and drain solutions are precisely controlled in real-time by an automated system of programmable four peristaltic pumps (fig. S73 and movie S1). When both source and drain are empty, no current flows, corresponding to the cutoff state, keeping the robot motionless. With 1 M NaCl solution on both sides, a low current occurs, also in the cutoff state. Changing the drain to 0.5 M MgCl_2_ solution (1 M NaCl solution in source) allows Na^+^ transport, generating a higher current above threshold 1, causing the robot to lift its left leg. Similarly, changing the source to 0.5 M Na_2_SO_4_ solution (0.5 M MgCl_2_ solution in drain) enables transport of both Cl^−^ and Na^+^, producing a current that reaches the saturation state, surpassing threshold 2, and prompting the robot to lift its right leg (fig. S75 and movie S1). Moreover, by controlling ion compositions, we defined low (Na^+^ only) and high (both Na^+^ and Cl^−^) current states, generating sequences of high and low current levels within short intervals (Fig. 4E). These encoded electrical signals could be subsequently collected and translated into readable character information using the American Standard Code for Information Interchange (ASCII) system (fig. S76) and used to instruct robotic arm motions (Fig. 4F). These experiments exemplify a unified energy-information flow framework inspired by biological systems, wherein the bidirectional ion transport in NP-MXene channels enables simultaneous osmotic energy conversion and signal transmission. The magnitude of osmotic output serves as a direct and quantifiable proxy for signal intensity, demonstrating coherent coupling between energy generation and information encoding for iontronic human-machine interaction.

It should be noted that these examples represent early applications of multi-ion transport in 2D nanofluidic channel materials. Looking ahead, Janus membranes like the NP-MXene structure could facilitate more complex multi-ion transport devices with additional carriers, advancing iontronic logic control and neuromorphic information processing. These channels can be easily created by restacking stripped 2D nanodiscs, providing scalability to meet future demands for device fabrication and applications.

## DISCUSSION

In summary, inspired by the ClC-5 ion channel, we fabricated NP-MXene channels with biomimetic dimensions, binding sites, and bidirectional ion permselectivity. Experimental and simulation results demonstrate that modifications with EDTA and PDDA enhance both ion selectivity and structural stability within the subnanochannels in MXene. The high surface charge density and hydrogen bonding in these channels contribute to excellent mono/divalent ion selectivity and permeability. For enhanced osmotic effects, the NP-MXene device achieved a record osmotic power density of 85.1 W m^−2^ and an exceptionally high membrane voltage of 181.5 mV under a 50-fold salinity gradient, the highest reported for a single device. Beyond power generation, these ion channels also enable information transmission, with salinity gradient–driven modulation of ion types achieving distinct cutoff, linear, and saturation states in iontronic transistors for robotic control without external gate voltage. These findings highlight the potential of multi-ion selective channels in complex fluid systems, paving the way for stable neuromorphic information processing, advanced 2D iontronic logic control, and scalable high-performance iontronics for future applications.

## MATERIALS AND METHODS

### Fabrication of NP-MXene ion channel membrane

The etching solution was prepared by adding 1 g of lithium fluoride (purchased from Macklin) to 20 ml of hydrochloric acid (12 M, purchased from Xilong Science Co. Ltd.), followed by stirring for 10 min. Then, 1 g of Ti_3_AlC_2_ powder (purchased from FoShan XinXi Technology Co. Ltd.) was slowly added to the above etching solution at 45°C and stirred for 48 hours. The acidic suspension was washed with deionized water using centrifugation at 3500 rpm for 5 min per cycle, and the centrifugal washing of a supernatant collected after each cycle was repeated until pH > 6. At around pH ≥ 6, a stable dark green supernatant of Ti_3_C_2_T*_x_* was observed, and then a final supernatant was collected after additional centrifugation at 3500 rpm for 60 min. EDTA-2Na solutions were prepared at a concentration of 1.5 mg ml^−1^. Specific amounts of Ti_3_C_2_T*_x_* dispersion were added to 50 mL EDTA-2Na solution, followed by continuous stirring at room temperature for 6 hours to obtain negatively charged MXene (N-MXene) nanosheets. The Ti_3_C_2_T*_x_*-EDTA MXene membranes were then fabricated by filtering the Ti_3_C_2_T*_x_*-EDTA mixture solution through a mixed cellulose ester membrane (0.22-μm pore size and a diameter of 50 mm). After the membrane surface had no solution, filtration was continued for 10 min to remove the residual solution in the interlayer spacing. The obtained membrane was air-dried at ambient conditions and could be easily detached from the support. The positively charged MXene (P-MXene) nanosheets were prepared by the dropwise addition of the PDDA aqueous solution (10 ml, 5 wt %) in to the colloidal solution of the pristine MXene (100 ml, 0.5 mg ml^−1^). Then, the mixture was magnetically stirred for 24 hours. The solution was centrifuged at 3500 rpm for 1 h. Subsequently, the obtained sediment was washed twice with deionized water, centrifuging at 3500 rpm for 1 hour each time. The final sediment was redispersed in deionized water and sonicated for 5 min to obtain positively charged MXene nanosheets. P-MXene was obtained by filtration using a method similar to that described above.

### Characterization

All electrochemical properties were carried out by electrochemical workstation (Multi Auto-lab M204). Morphologies of MXene were observed by SEM (SU8020, Hitachi) with a 5.0-kV accelerating voltage and 10-μA emission current, and EDS was used for the element analysis. Dektak XT stylus profiler (Bruker) was used to profile the morphology of the membranes and provided information on areal values for the calculation of power density. Raman spectra were collected with the LabRAM HR Evolution (Horiba) with a 532-nm laser. FTIR spectra were collected by Bruker VERTEX80v. The ζ potential was obtained by using the DanDong Better instrument (BeNano Zeta). XRD was tested with a Bruker D8 ADVANCE system with Cu Kα source. Particle size analysis was measured with the Bettersize2600 instrument. Atomic force microscopy (AFM) images of MXene nanosheets were taken by a Dimension Icon AFM (Bruker AXS, Germany) under ambient conditions. The XPS measurements have been conducted on a Thermo Fisher Scientific K-Alpha, in a vacuum of 1 × 10^−9^ mBar, using an Alka ray source (hv = 1486.6 eV), the working voltage is 15 kV and the filament current is 10 mA. Ion chromatograph (Dionex Aquion IC, ICS-600) was used for determining of ion concentrations in permeates. TEM and elemental mapping images were obtained using a transmission electronmicroscope (Thermo Fisher Scientific–AQUION USA, Thermo Fisher Scientific–Tecnai G2 F30 USA). To obtain the membrane cross-sectional TEM images, the membrane was first cut into 3 mm by 5 mm rectangle by blade and then used Epon812 resin for embedding. The embedded membrane samples were processed by an ultrathin microtome (Leica-EM UC 7, Germany), and lastly, an ultrathin resin sheet exposing the section of the membrane samples was obtained, with a thickness about 50 nm.

### Electrical measurement

The membrane samples were mounted between two electrolyte cells. Each cell was filled with 200 μl of solution (KCl, MgCl_2_, or Na_2_SO_4_). Varied concentrations of solution were sequentially applied at the two sides of the 2D nanofluidic channels, constructing the salinity gradient. The *IV* curves of the N-MXene, P-MXene, and NP-MXene osmotic power sources were recorded by electrochemical workstation (Multi Auto-lab M204). To evaluate the effect of electrode potential on electrical measurement, a pair of Ag/AgCl standard electrodes were used to apply the transmembrane potential and measure the resulting current-voltage responses of the tested N-MXene, P-MXene, and NP-MXene osmotic power sources. The intercept on the vertical axis (*I*_SC_) represents the net osmotic current. Correspondingly, the intercept on the horizontal axis (*V*_OS_) represents the osmotic potential. For basic output performance testing of the NP-MXene iontronic transistor, a programmable electrostatic voltmeter (Keithley 6514) was directly connected to a synchronized data acquisition card (National Instruments 6346) to measure current signals.

## Supplementary Materials

### The PDF file includes:

Supplementary Text S1 to S4 Figs. S1 to S76 Tables S1 to S4 Legend for movie S1 References
