# Supplementary material for: Biomimetic Janus MXene membrane with bidirectional ion permselectivity for enhanced osmotic effects and iontronic logic control
Source: Sci Adv. 2025 Sep 17;11(38):eadx1184. doi: 10.1126/sciadv.adx1184 (PMC12442869; doi:10.1126/sciadv.adx1184)
Supplement: Supplementary file 1 — Supplementary Text S1 to S4 Figs. S1 to S76 Tables S1 to S4 Legend for movie S1 References [file sciadv.adx1184_sm.pdf]

Science Advances
AAAS

Supplementary Materials for

Biomimetic Janus MXene membrane with bidirectional ion permselectivity for enhanced osmotic effects and iontronic logic control

Han Qian *et al.*

Corresponding author: Yanguang Zhou, maeygzhou@ust.hk; Zhong Lin Wang, zhong.wang@mse.gatech.edu; Di Wei, dw344@cam.ac.uk

Sci. Adv. **11**, eadx1184 (2025)
DOI: 10.1126/sciadv.adx1184

The PDF file includes:

Supplementary Text S1 to S4
Figs. S1 to S76
Tables S1 to S4
Legend for movie S1
References

Other Supplementary Material for this manuscript includes the following:

Movie S1

Supplementary Text

Supplementary Text 1

Ion permeation measurement

The H-shaped diffusion setup with two 30 mL diffusion reservoirs was used for membrane ion permeation measurement (fig. S32). The NP-MXene membrane was sealed between acrylic plates with rectangular openings using epoxy resin, with silicone gaskets on both sides, and then clamped in the center of the H-shaped diffusion apparatus. The solution in the feed chamber (C_1) was a mixed solution of 0.5 M $MgCl_2$ and 0.5 M Na_2SO_4, while the permeate chamber (C_2) contained DI water. After diffusion, the ion concentrations in the solutions on both sides were measured using ion chromatography (DIONEX AQUION, ICS-600).

The ion permeation rate P_i (mol m^{-2} h^{-1}) calculation is given by:

$$P_i = \frac{(C_A - C_B)\cdot V}{A\cdot t} \quad (1)$$

Where C_A (mol L^{-1}) is the ion concentration after diffusion, C_B (mol L^{-1}) is the corresponding ion concentration before diffusion, V (L) is the volume of the solution on the permeation side, A is the effective membrane area (3×10^{-8} m^2), and t (h) is the diffusion time.

Membrane ion selectivity, S, is calculated as follows:

$$S = \frac{P_{i1}/C_{i1}}{P_{i2}/C_{i2}} \quad (2)$$

Where P_{i1} (mol m^{-2} h^{-1}) is the permeation rate of ion 1, and C_{i1} (mol L^{-1}) is the concentration of ion 1 in the feed solution (mol L^{-1}).

Supplementary Text 2

Ion selectivity of NP-MXene osmotic power sources

Due to the cation-selective and anion-selective nature of the N-MXene and P-MXene sub-nanochannels, respectively, it is possible to preferentially transport ions from the high-concentration side to the low-concentration side, generating the membrane potential (V_{OS}). The V_{OS} originates from the ion selectivity of the N-MXene and P-MXene sub-nanochannels, which can result in differences in the diffusive fluxes of different anions and cations. The cation transference number (t_+) or antion transference

number (t_-) are calculated following equations:

$$t_n = \frac{1}{2}\left(\frac{V_{OS}}{\frac{RT}{zF}ln\frac{\lambda_{C_H}c_H}{\lambda_{C_L}c_L}} + 1\right) \quad (3)$$

Where V_{OS} refers to the osmotic potential; R, T, Z, and F refer to the gas constant, temperature, valence charge, and Faraday constant, respectively; λ and c refer to the ion activity coefficient and concentration.

Supplementary Text 3

Molecular dynamics simulations

To further illustrate the dynamic transport behaviours of various ions in the 2D nanofluidic channels, we additionally performed molecular dynamics (MD) simulations to obtain atomic-level insights into ionic transport in the 2D nanofluidic channels. The MD simulation was implemented by the software of Large-scale Atomic/Molecular Massively Parallel Simulator (LAMMPS) (*65*). In the MD simulation, the simulation model of NP-MXene was constructed as shown in Figure S37. For the feasibility and convenience of simulations, the ionic transport channels in the experiment were simplified as the gap formed by two layers of stacked MXene layers and the length and width of each MXene layer was 70 and 19.6 Å, respectively. To model the MXene layers, the MXene layer was functionalized with hydroxyl (-OH) functional groups, which were uniformly distributed on both sides of the layer. The height of nanochannels between two stacked MXene layers was set according to the experimental measured value. The actual width of the nanochannel is 10 Å, while the equilibrium width of the interaction is approximately 3.7 Å. Four stacked MXene layers were used to construct the MD model. To simulate the ionic transport through the 2D nanofluidic channels, the one side space was filled with different salt solutions (KCl, $MgSO_4$, $MgCl_2$, Na_2SO_4), and the other side space was filled with pure water at the initial state. The concentration of each salt solution was ~1 M.

In MD simulations, the water model of TIP4P/2005 model was selected to describe the water molecules (*66*). To model the positively charged and negatively charged nanochannles of MXene membranes, the EDTA and PDDA functionalized nanochannels are simplified. Th atomic charge of O atoms in the hydroxyl (-OH)

functional groups are set as 0.1 (positively charged) and -0.1 (negatively charged). The atomic charges of other atoms are ignored to make charge equilibrium of the whole system. The interaction parameters of atoms in MXene were extracted from the Universal force field (*67*). The interactions between water molecules and different ions (K^+, Na^+, Mg^{2+}, Cl^-, and SO_4^{2-}) and the interactions among the different ions were described by the Lennard-Jones 6-12 potential, and the interaction parameters from Ref. (*68*) were adopted. The cut-off distance of the van der Waals interactions was 12 Å. The long-range electrostatic interactions were considered in MD simulations. The particle-particle particle-mesh (PPPM) solver with a relative force error of 10^{-5} was employed to solve the long-range electrostatic interactions. The time step of MD simulation was 2 fs. The Nose Hoover thermostat was used to control the temperature of salt solution. Periodical boundary conditions were applied to x and y directions. To prevent the leakage of salt solution, the graphene wall and vacuum layer with a length of 10 Å were added at the two ends of model. The temperature of salt solution was controlled to be 500 K. It should be noted that the higher temperature was mainly for accelerating the ionic movement in simulations since the simulation time was limited for MD method.

Supplementary Text 4

Density functional theory (DFT) calculation

Density Functional Theory calculations (DFT) employed the Generalized Gradient Approximation (GGA) parameterized by the Perdew-Burke-Ernzerhof (PBE) formula to evaluate electron exchange-correlation energy. Structural parameters and all atoms were fully optimized. The wave function expansion was truncated at 500 eV, and the energy convergence criterion was set to 10^{-6} eV to ensure high precision in the calculations.

Supplementary Figures

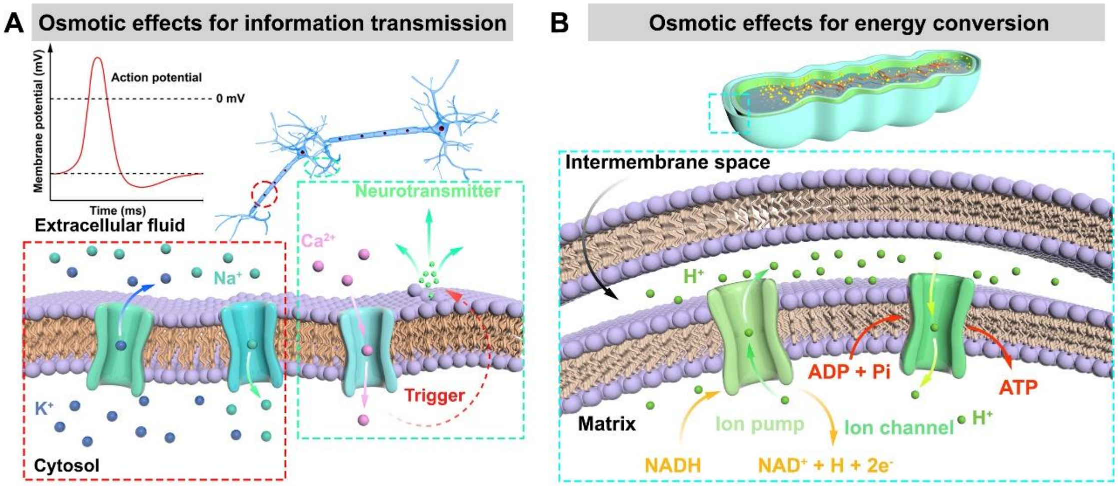

Fig. S1. Energy-information flow in biological systems. (**A**) Osmotic effects as information flow in nerve impulses. (**B**) Osmotic effects are involved in ATP synthesis in mitochondria (energy conversion via chemiosmotic coupling).

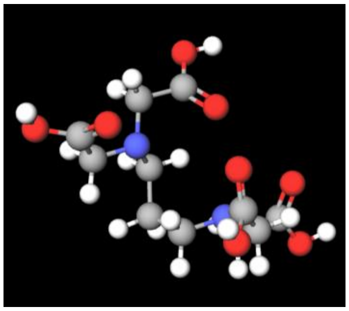

Fig. S2. Structure of EDTA molecule.

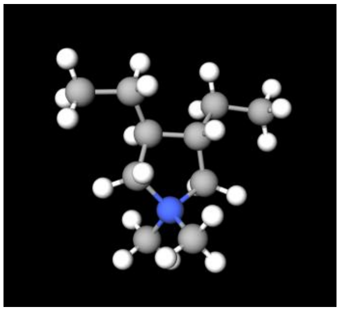

Fig. S3. Structure of PDDA molecule.

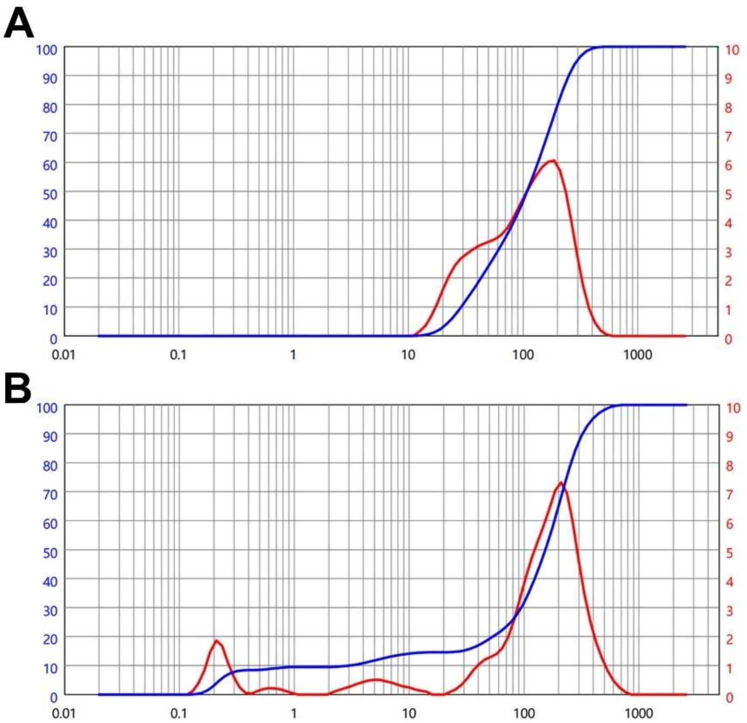

Fig. S4. The particle size distribution of (A) N-MXene and (B) P-MXene nanosheets (x-axis: μm, y-axis: %).

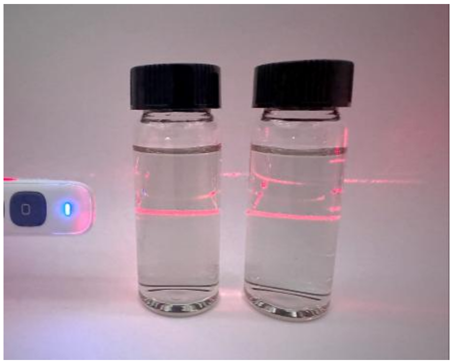

Fig. S5. The Tyndall effect of N-MXene (left) and P-MXene (right) solutions, which demonstrates the uniform dispersion of the nanosheets.

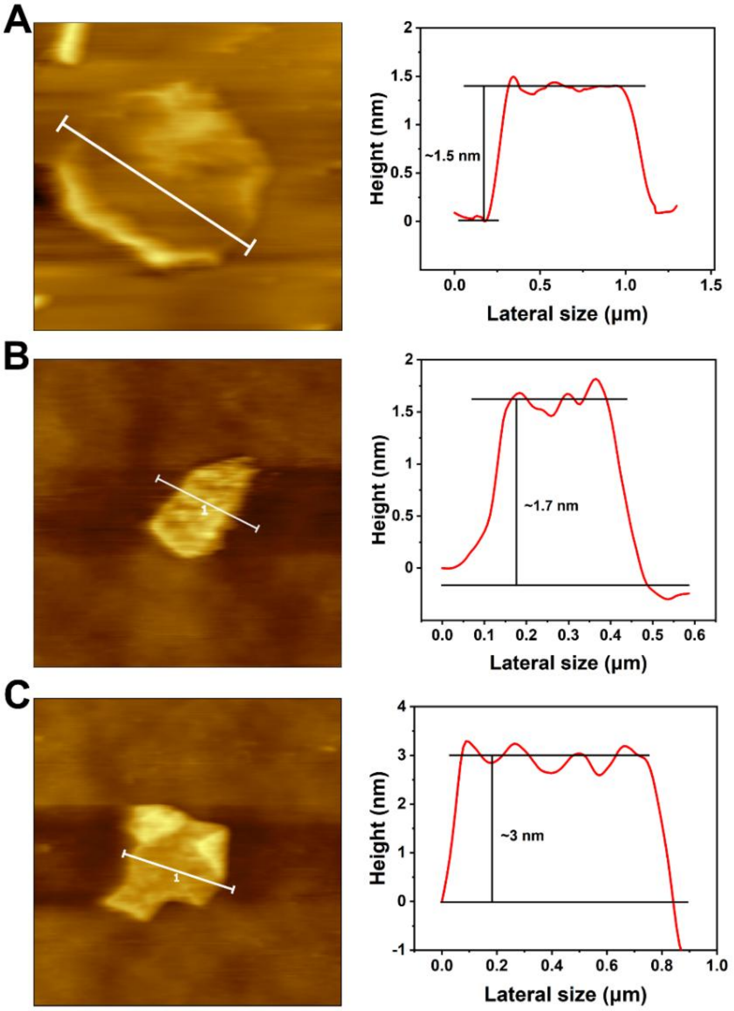

Fig. S6. AFM images and corresponding height profiles the (A) MXene, (B) N-MXene, and (C) P-MXene nanosheets.

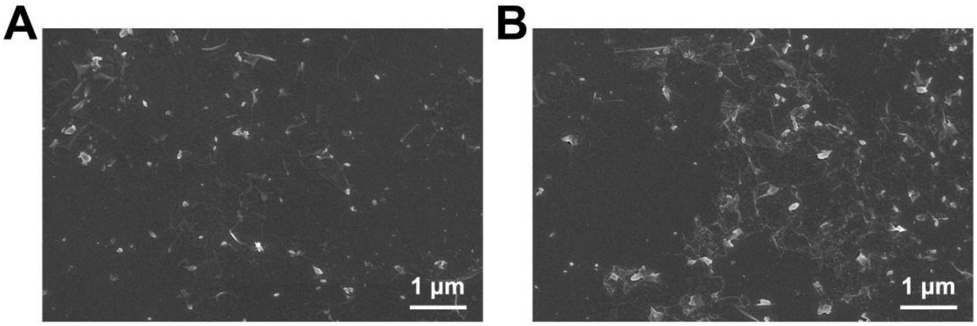

Fig. S7. SEM images of the (A) N-MXene and (B) P-MXene nanosheets supported on the silicon wafer.

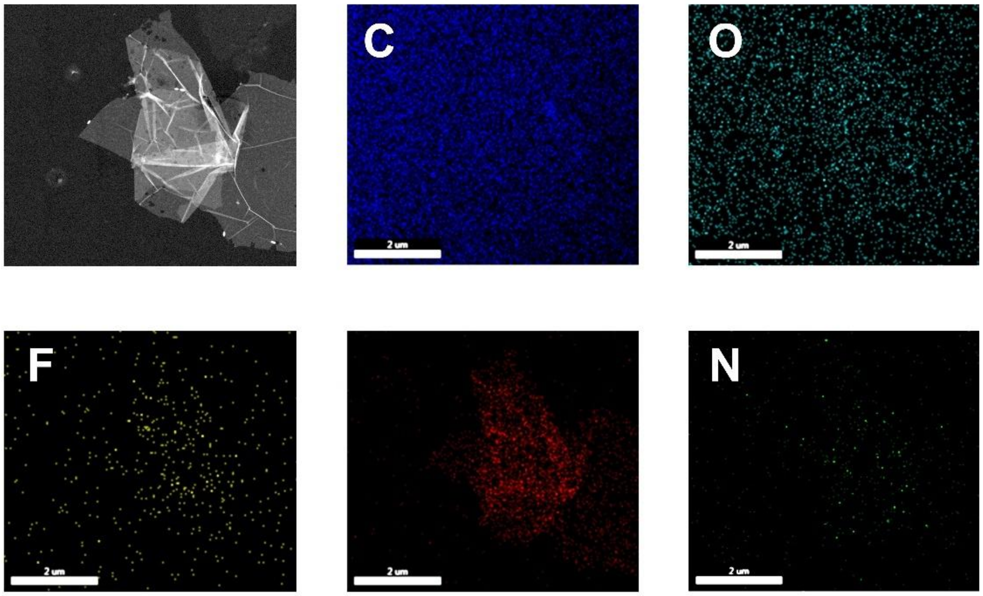

Fig. S8. TEM image and the corresponding elemental mappings of the MXene nanosheets.

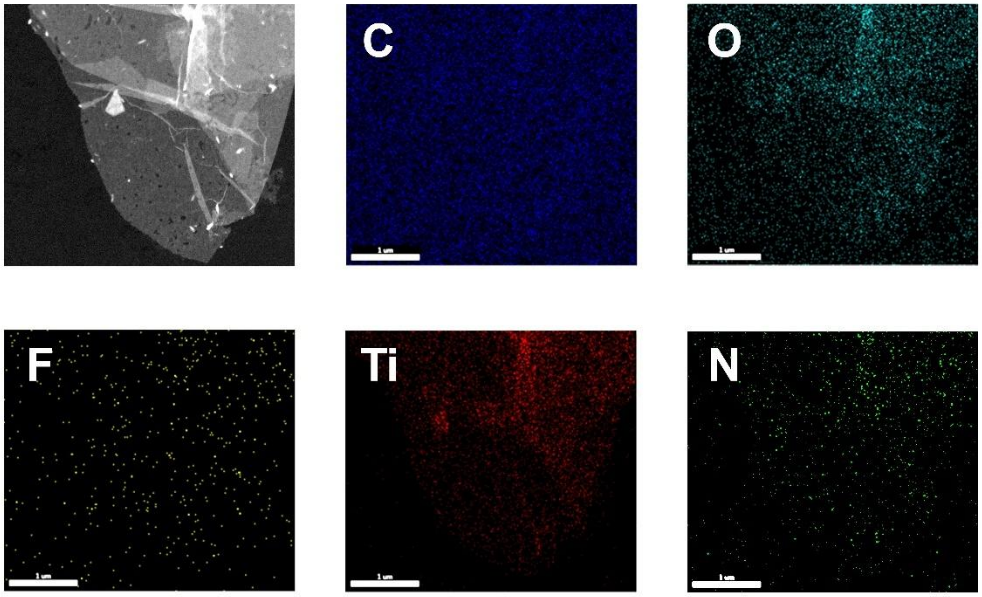

Fig. S9. TEM image and the corresponding elemental mappings of the N-MXene nanosheets.

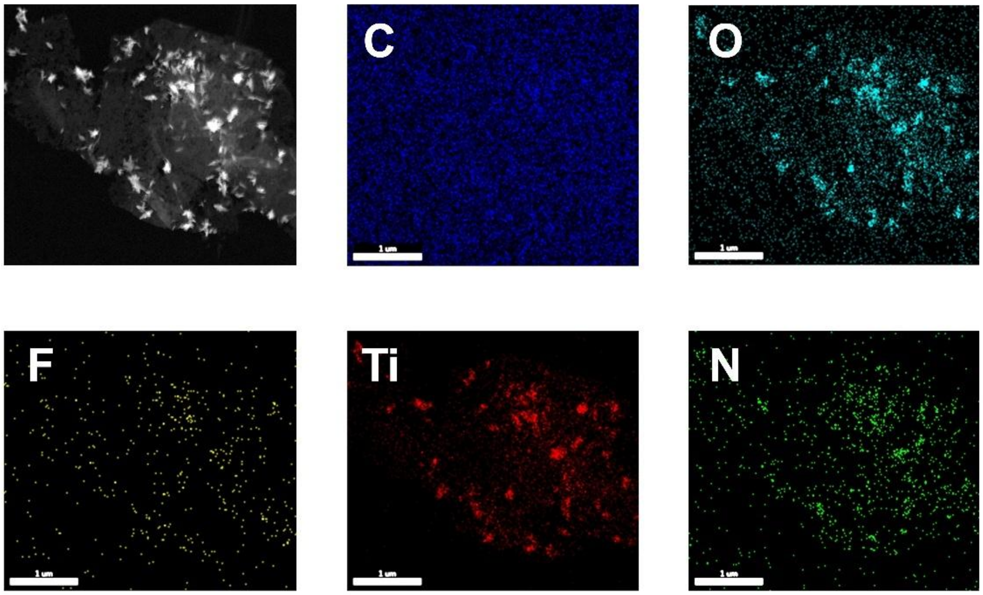

Fig. S10. TEM image and the corresponding elemental mappings of the P-MXene nanosheets.

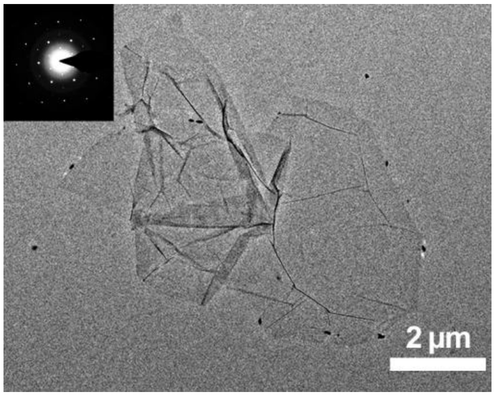

Fig. S11. TEM image and selected area electron diffraction (SAED) pattern of the exfoliated MXene nanosheet.

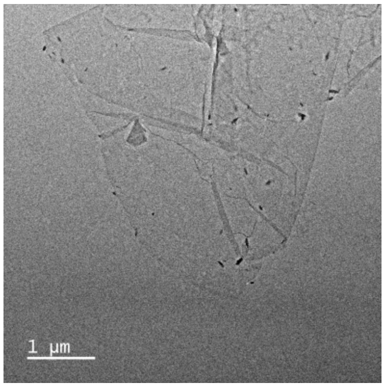

Fig. S12. TEM image of the exfoliated N-MXene nanosheet.

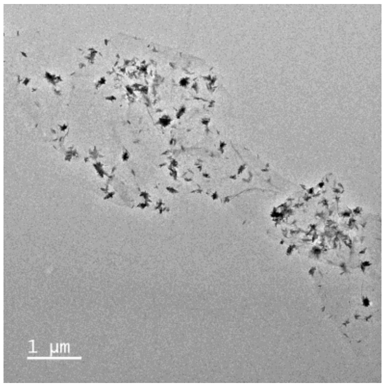

Fig. S13. TEM image of the exfoliated P-MXene nanosheet.

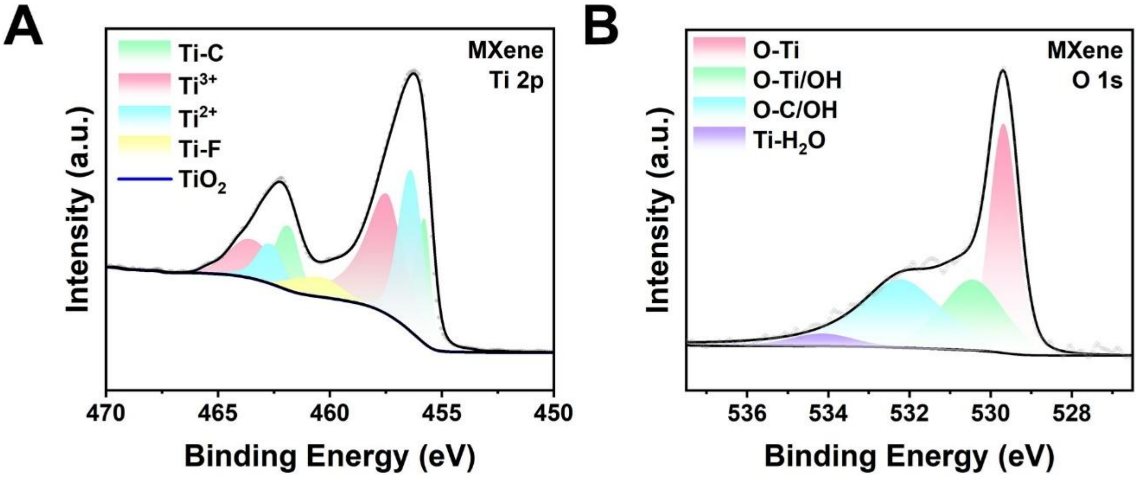

Fig. S14. XPS high-resolution (A) Ti 2*p* and (B) O 1*s* XPS spectra of as-synthesized $Ti_3C_2T_x$ nanosheets.

Four surface terminations of $Ti_3C_2T_x$ nanosheets were obtained using LiF and HCl etching methods: Ti–O (type I), Ti–OH (type II), Ti–F (type III), and Ti–$(H_2O)_{ads}$ (type IV) (*69*). In the Ti 2p spectra, the main peak of Ti $2p_{3/2}$ can be fitted with five peaks: 455.8 eV (Ti–C), 456.4 eV (Ti^{2+} for types I, II, and IV), 457.5 eV (Ti^{3+} for types I, II, and IV), 458.2 eV (TiO_2), and 460.5 eV (Ti–F for type III) (*70*). The peak intensity of TiO_2 is almost negligible, indicating that the nanosheets were not oxidized during synthesis. In the O 1s spectra, the main peak can be fitted with four peaks at 529.7 eV (Ti–O), 530.4 eV (Ti–OH), 532.2 eV (C–O), and 534.1 eV (Ti–H_2O). Lewis acid Ti sites, which have empty orbitals, are common on the surface of $Ti_3C_2T_x$ MXene and are temporarily occupied by water molecules to form Ti–H_2O in aqueous solutions (*35, 42, 70*).

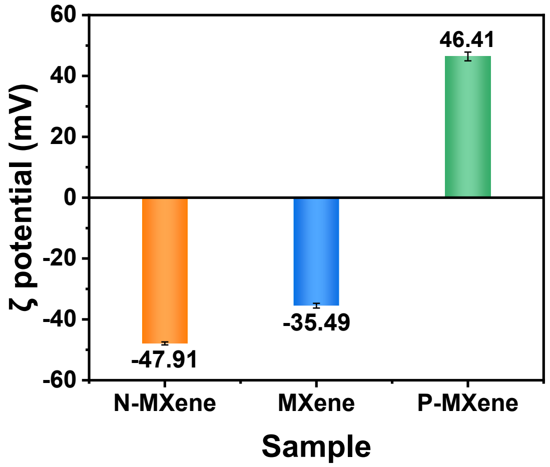

Fig. S15. Zeta potential of the MXene, N-MXene, and P-MXene dispersions.

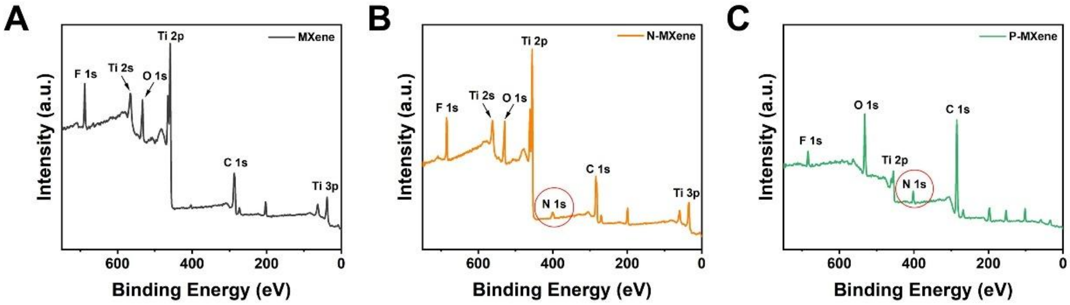

Fig. S16. XPS survey spectra of (A) MXene, (B) N-MXene, and (C) P-MXene membrane.

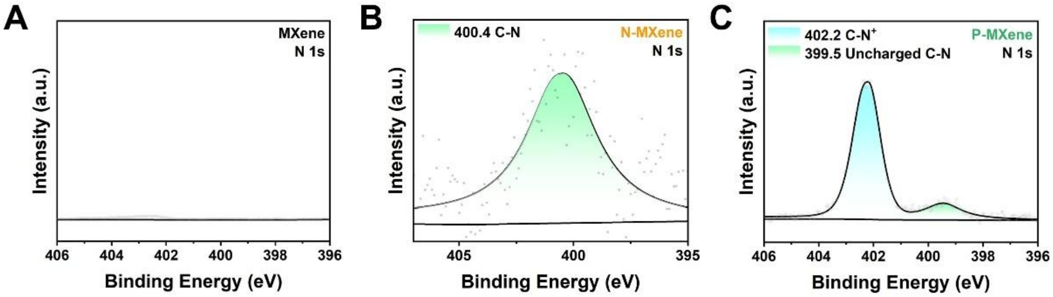

Fig. S17. N 1*s* XPS spectra of (A) MXene, (B) N-MXene, and (C) P-MXene membrane. For the MXene, no obvious peak is observed in the N 1s region. In contrast, for the P-MXene, the fitted peaks at ~402.2 eV and ~399.5 eV correspond to charged and uncharged quaternary amine moieties.

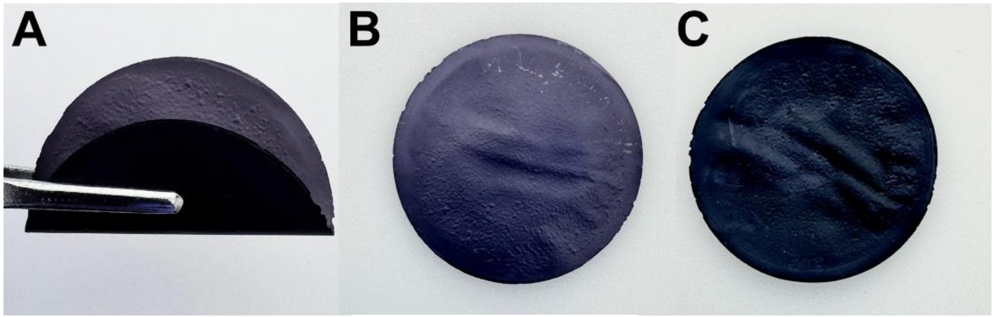

Fig. S18. Optical photos of different films. (A) Freestanding NP-MXene membrane in a bending state. **(B and C)** Photographs of the two sides of the NP-MXene membrane (N-MXene side and P-MXene side), showing the difference in color.

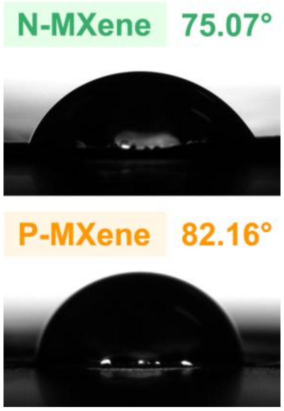

Fig. S19. Contact angle of the NP-MXene. The two sides of the NP-MXene are hydrophilic, with water contact angles of 75.07° for the N-MXene side and 82.16° for the P-MXene side.

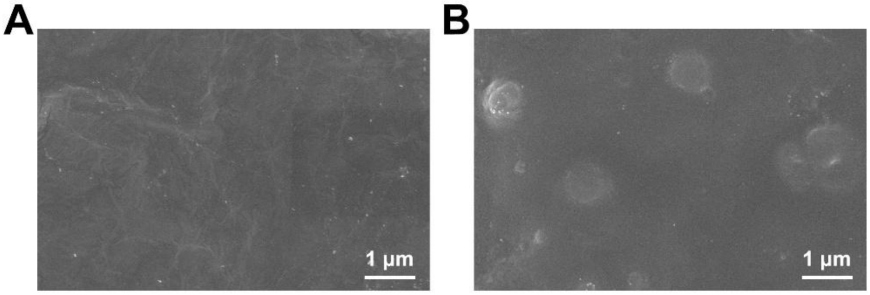

Fig. S20. SEM images of the surface of the (A) N-MXene and (B) P-MXene layer.

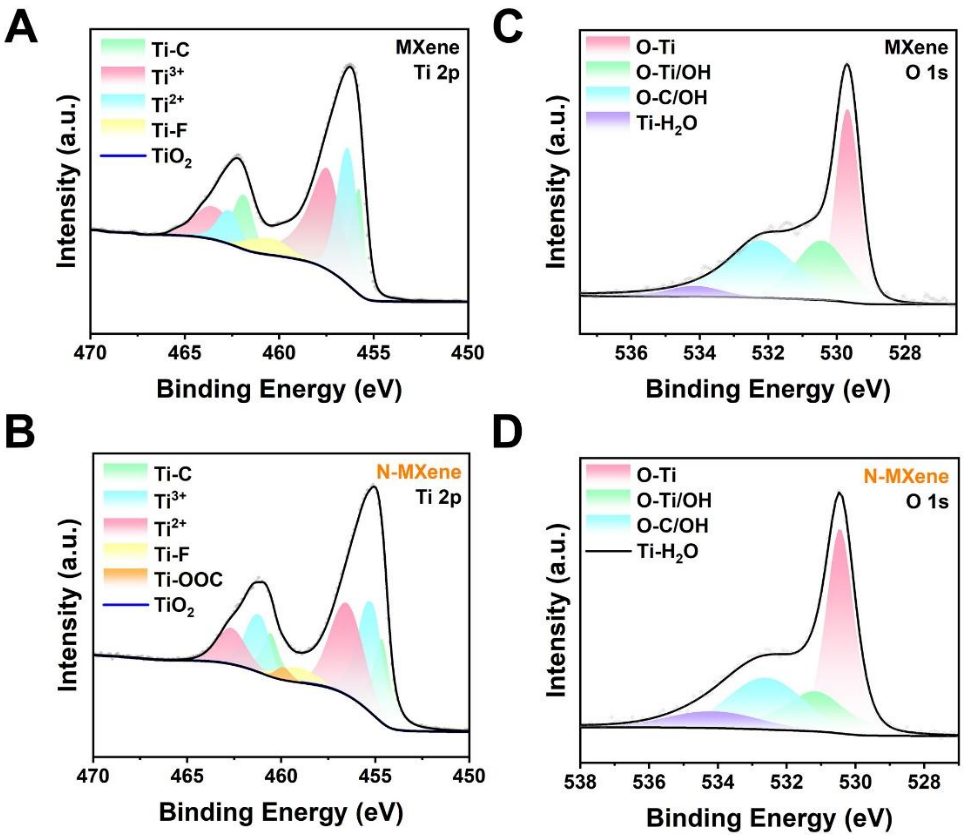

Fig. S21. (A) Ti 2*p* XPS spectra of MXene membrane. (B) Ti 2*p* XPS spectra of N-MXene membrane. (C) O 1*s* XPS spectra of MXene membrane. (D) O 1*s* XPS spectra of N-MXene membrane.

Four surface terminations of $Ti_3C_2T_x$ nanosheets were obtained using LiF and HCl etching methods: Ti–O (type I), Ti–OH (type II), Ti–F (type III), and Ti–(H_2O)ads (type IV) (*69*). In the Ti 2*p* spectra, the main peak of Ti $2p_{3/2}$ can be fitted with five peaks: 455.8 eV (Ti–C), 456.4 eV

(Ti^{2+} for types I, II, and IV), 457.5 eV (Ti^{3+} for types I, II, and IV), 458.2 eV (TiO_2), and 460.5 eV (Ti–F for type III) (*70*). The peak intensity of TiO_2 is almost negligible, indicating that the nanosheets were not oxidized during synthesis. After modification with EDTA molecules, the covalent bonds between oxygen atoms and titanium atoms, which generally have higher electronegativity, lead to a decrease in the outer electron cloud density of the titanium atoms. This reduction in electron cloud density weakens the shielding effect (*71*), causing the Ti-C $2p_{3/2}$ and Ti^{2+} (I, II, IV type) $2p_{3/2}$ peaks to shift from 455.8 eV and 456.4 eV in the MXene membrane to 454.7 eV and 455.3 eV in the N-MXene membrane. Additionally, a peak appears at 460 eV due to the coordination between titanium atoms and the carboxyl groups in the EDTA molecules (*35*). In the O 1s spectra of MXene, the main peak of O 1s can be fitted by four peaks at 529.7 eV (Ti–O), 530.4 eV (Ti–OH), 532.2 eV (C–O), and 534.1 eV (Ti–H_2O). After modification with EDTA molecules, the Ti–O, Ti–OH and C–O peaks are upshifted from 529.7, 530.4 and 532.2 eV for MXene to 530.4, 531.2 and 532.6 eV for N-MXene, respectively. These changes indicate that Ti–O and Ti– OH are the active sites of EDTA molecules (*35*).

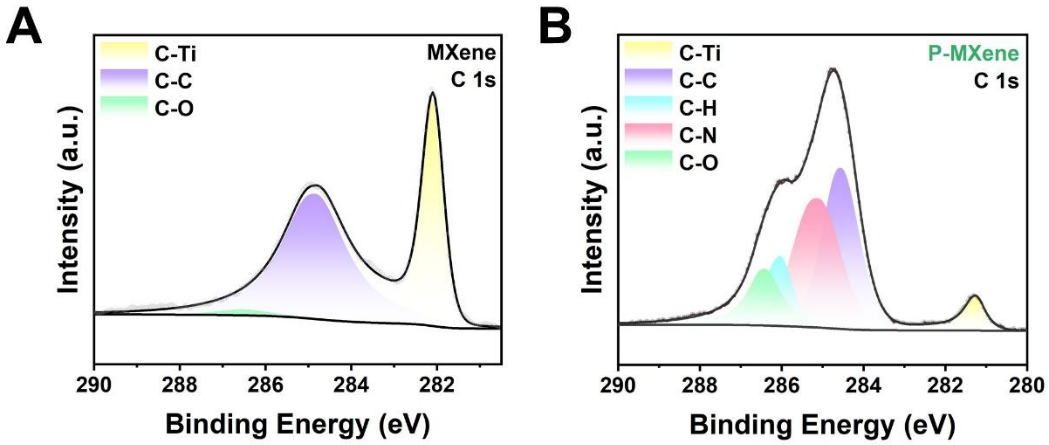

Fig. S22. (A) C 1*s* XPS spectra of MXene membrane. (B) C 1*s* XPS spectra of N-MXene membrane.

In the C 1*s* spectra of MXene, the main peak of C 1*s* can be fitted by three peaks at 282.1 eV (C–Ti), 284.7 eV (C–C), and 286.5 eV (C–O). After modification with PDDA molecules, the C 1s spectra of P-MXene membrane shows new C-N and C-H peaks at 285.1 eV and 282.1 eV, respectively, confirming the successful modification of MXene nanosheets with PDDA (*56*).

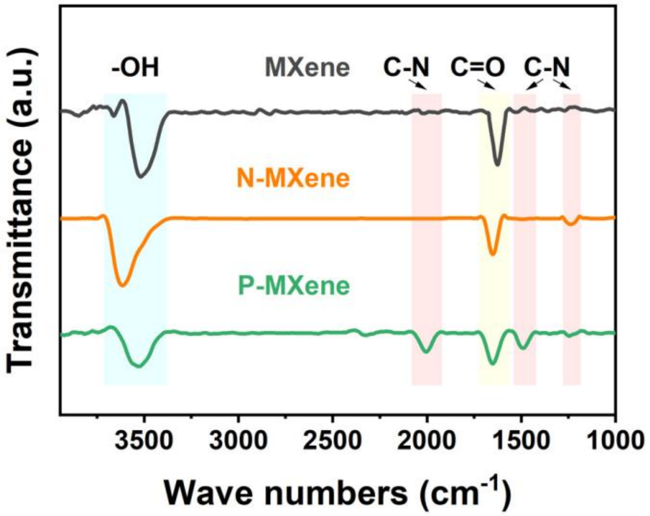

Fig. S23. FTIR spectrum of the N-MXene layer and P-MXene layer, the marked characteristic peaks correspond to the functional groups. FT-IR spectrum shows that, compared with MXene, N-MXene and P-MXene exhibit new peaks corresponding to the antisymmetric stretching vibration mode of C−N (*72, 73*), indicating the presence of EDTA and PDDA molecules. The peak for stretching vibration of –OH is redshifted, confirming the formation of hydrogen bonds (*71*). The decrease in C=O and -OH in the P-MXeen layer indicates that PDDA is adsorbed on the surface of the MXene by electrostatic attraction under the formation of hydrogen bonds (*56, 74, 75*).

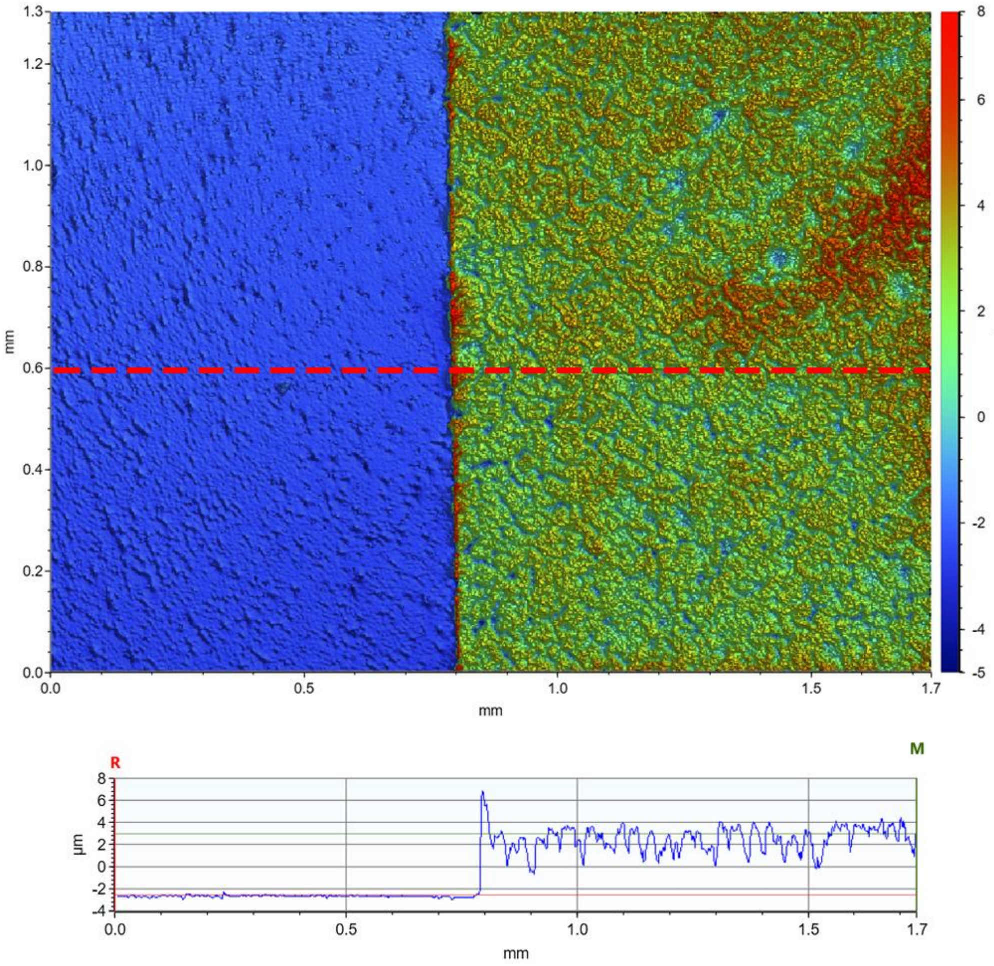

Fig. S24. Surface profile of the NP-MXene membrane (The thickness of NP-MXene membrane is ~5 µm).

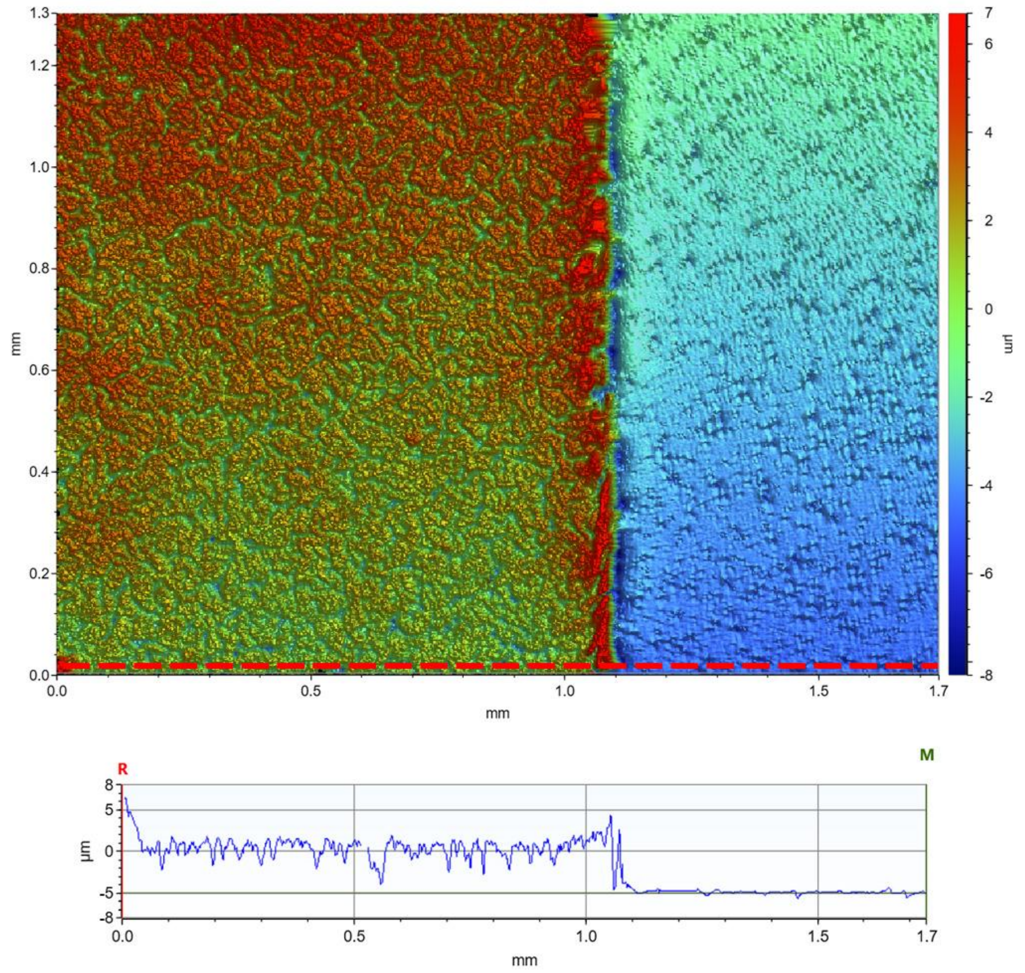

Fig. S25. Surface profile of the N-MXene membrane (The thickness of N-MXene membrane is ~5 µm).

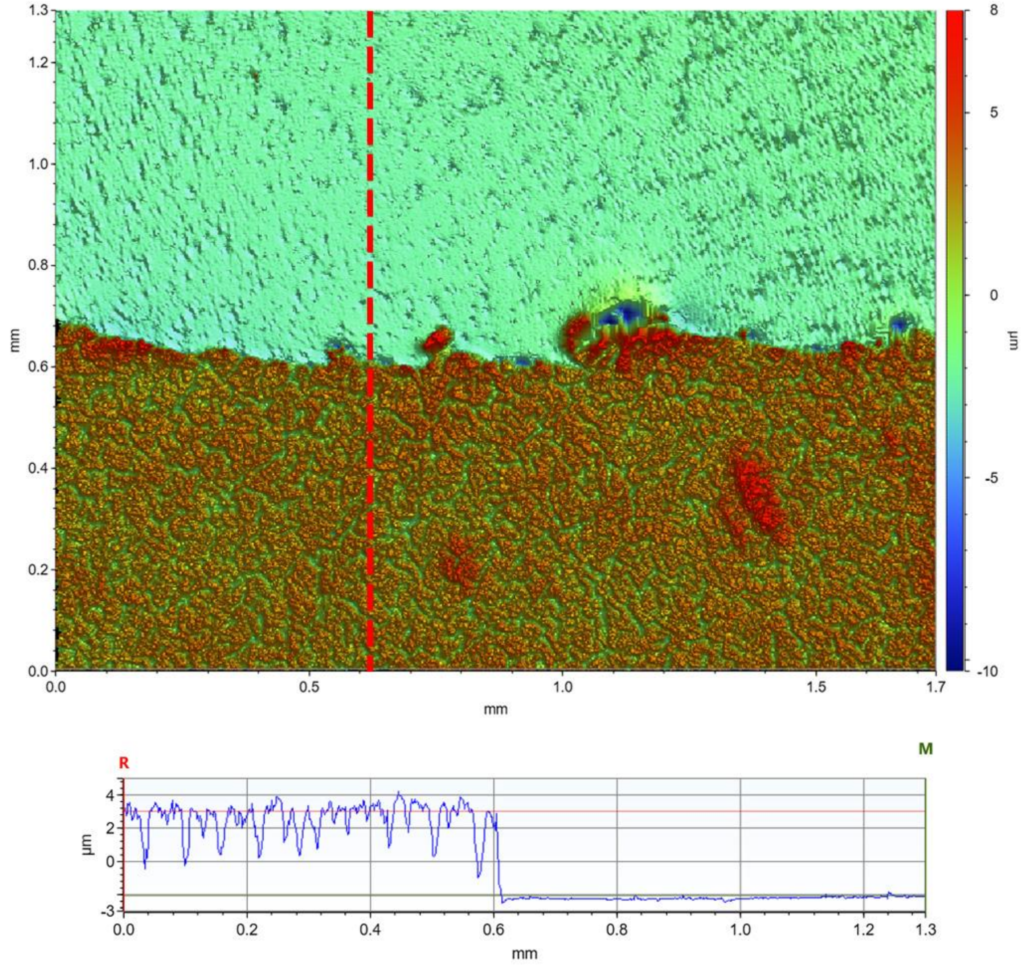

Fig. S26. Surface profile of the P-MXene membrane (The thickness of P-MXene membrane is ~5 µm).

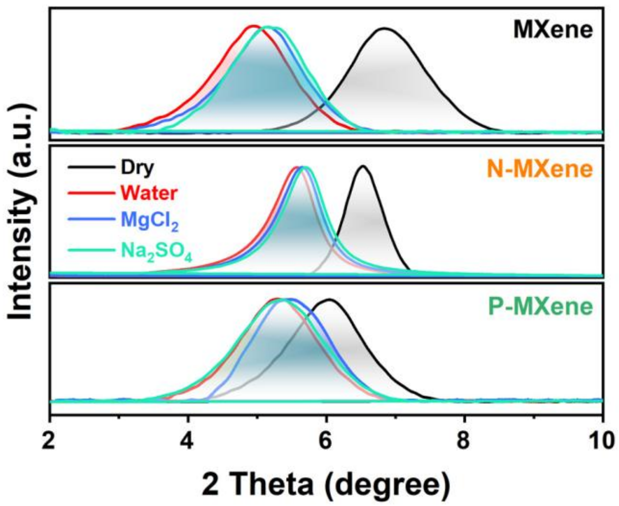

Fig. S27. XRD patterns of the MXene, N-MXene, and P-MXene membranes in the dry state and various solutions.

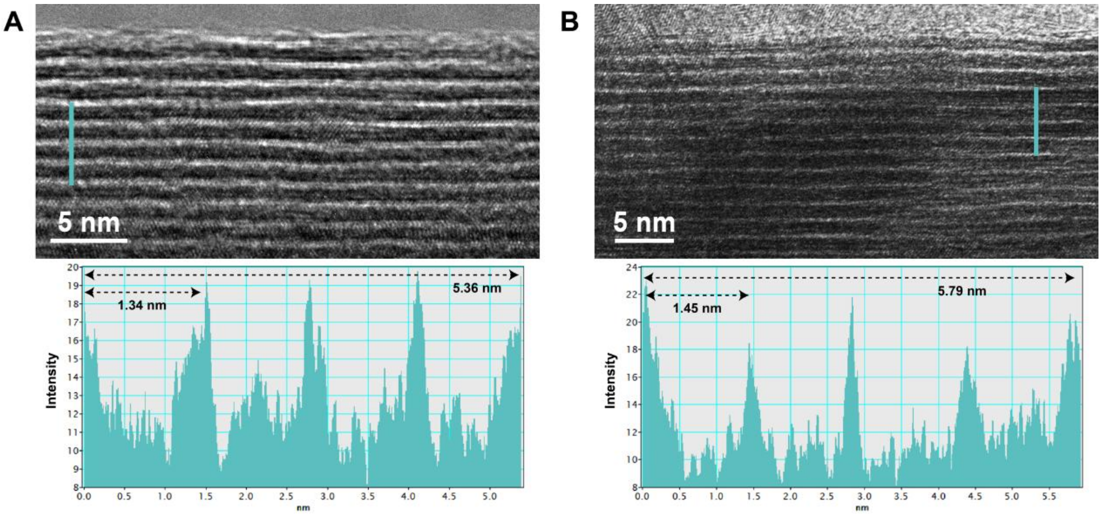

Fig. S28. High-resolution transmission electron microscopy (HRTEM) image of the cross-section of (A) N-MXene layer and (B) P-MXene layer in NP-MXene channels and the corresponding interlayer spacing between neighboring nanosheets taken along the line.

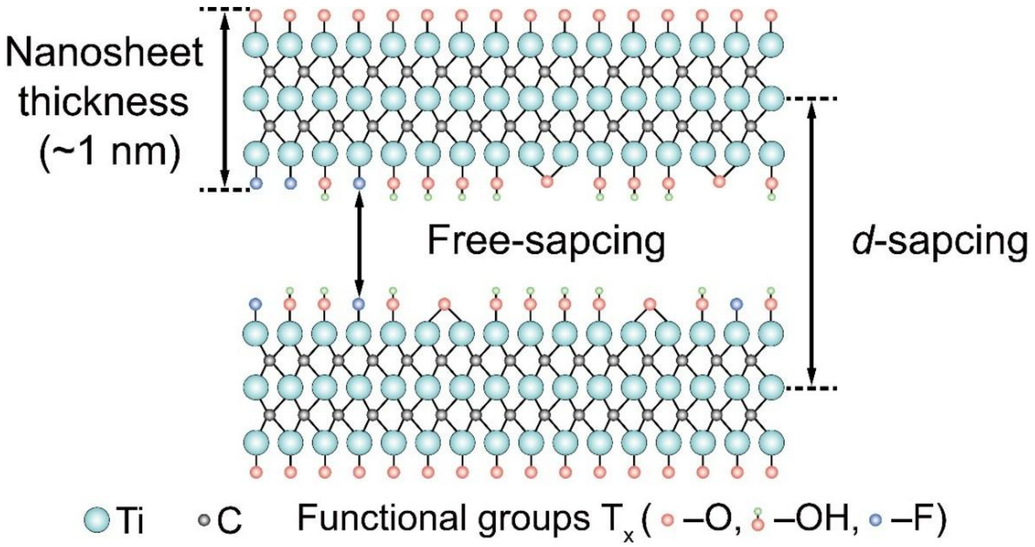

Fig. S29. Schematic diagram of free spacing calculation for NP-MXene Janus membrane. The d-spacing of the MXene Jauns permselective membranes were calculated using the [002] peak position in the XRD pattern, following Bragg's law (*76*). The *d*-spacing obtained by this method includes one $Ti_3C_2T_x$ layer and one free spacing, where the monolayer $Ti_3C_2T_x$ nanosheet has the thickness of 10.0 Å (*35, 37*).

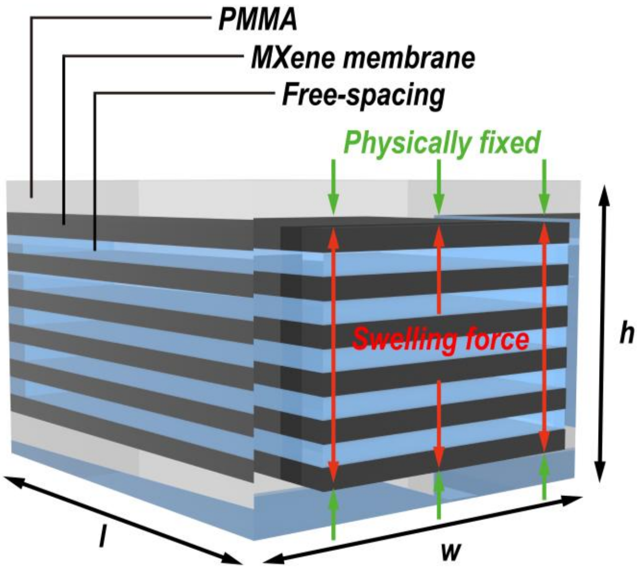

Fig. S30. Schematic of physically confined sub-nanochannel by PMMA (length *l* = 3 mm, width *w* = 5 mm, and height *h* = 5 μm).

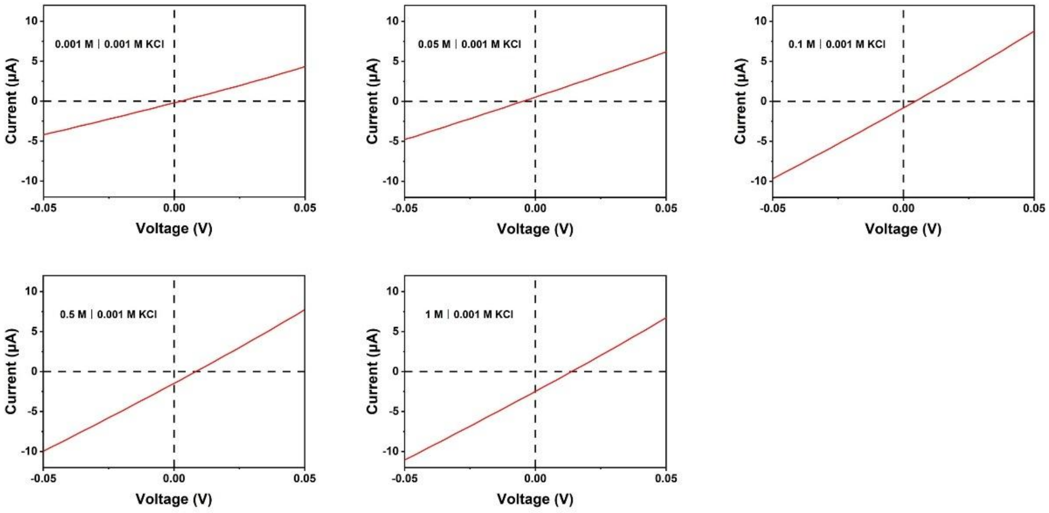

Fig. S31. ***IV* curves of NP-MXene membrane recorded in different KCl salinity gradient ratios of 10, 50, 100, 500 and 1000.**

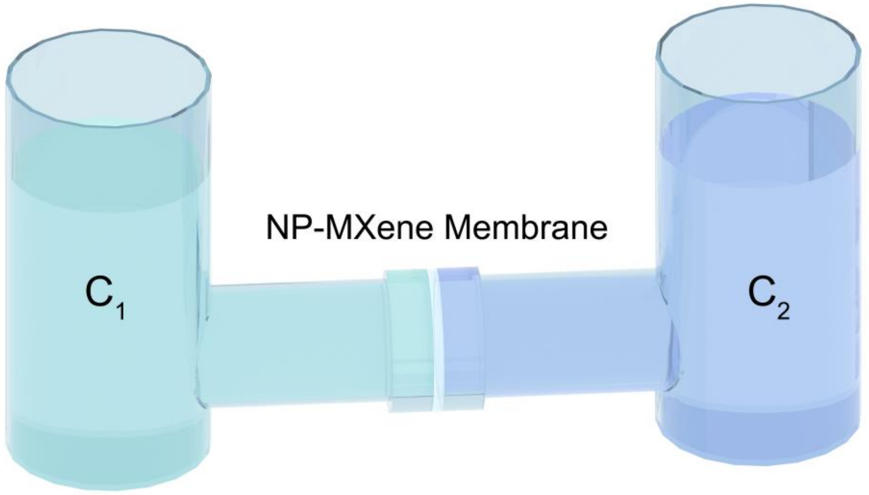

Fig. S32. Schematic of the H-shaped setup used for ion permeation measurements. An encapsulated membrane was fixed between feed chamber (C_1) and permeate chamber (C_2). The solution in the feed chamber (C_1) was a mixed solution of 0.5 M $MgCl_2$ and 0.5 M Na_2SO_4, while the permeate chamber (C_2) contained DI water.

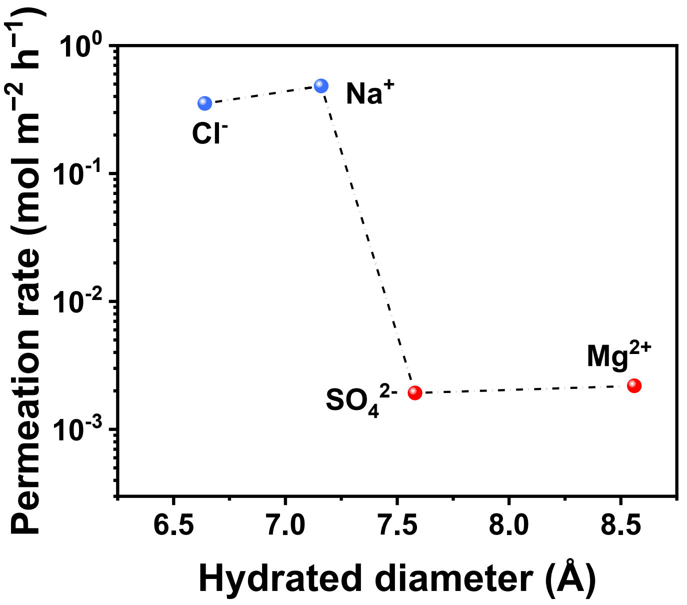

Fig. S33. The permeation rates through NP-MXene channels.

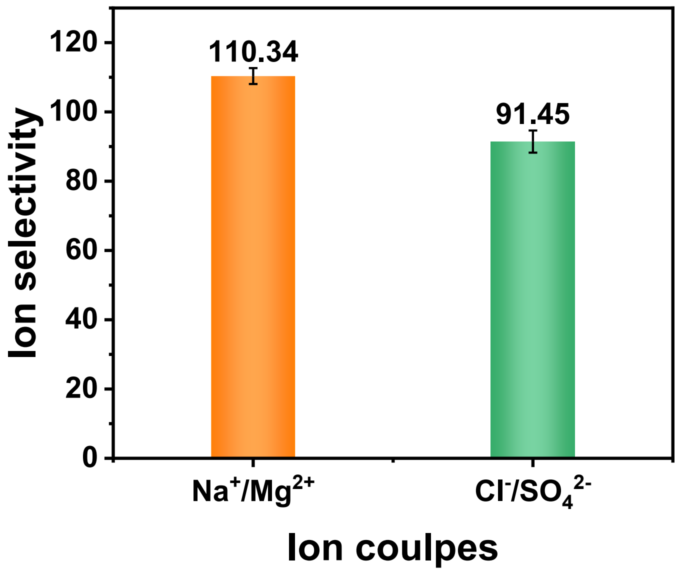

Fig. S34. The Na^{+}/Mg^{2+} and Cl^{-}/SO_4^{2-} selectivity of NP-MXene channels.

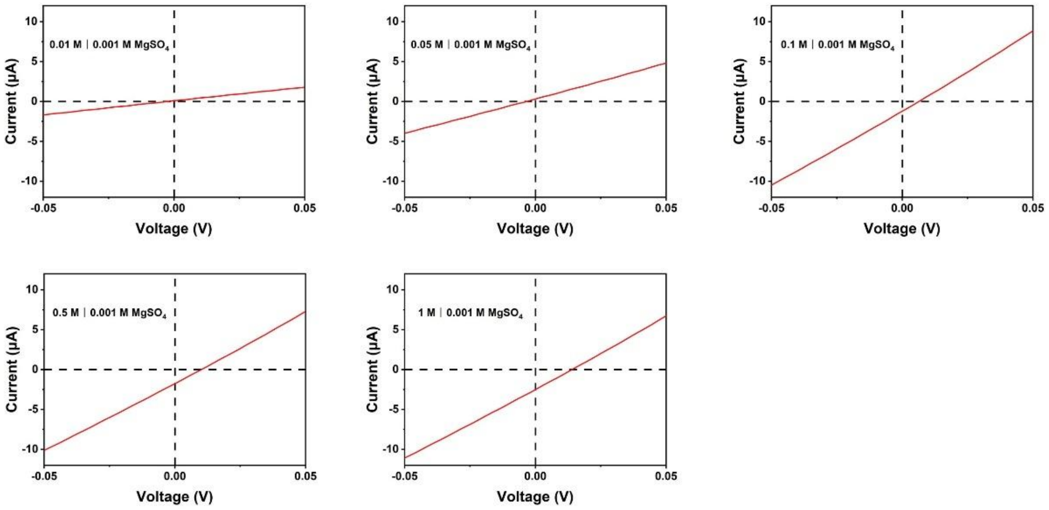

Fig. S35. ***IV*** **curves of NP-MXene membrane recorded in different $MgSO_4$ salinity gradient ratios of 10, 50, 100, 500 and 1000.**

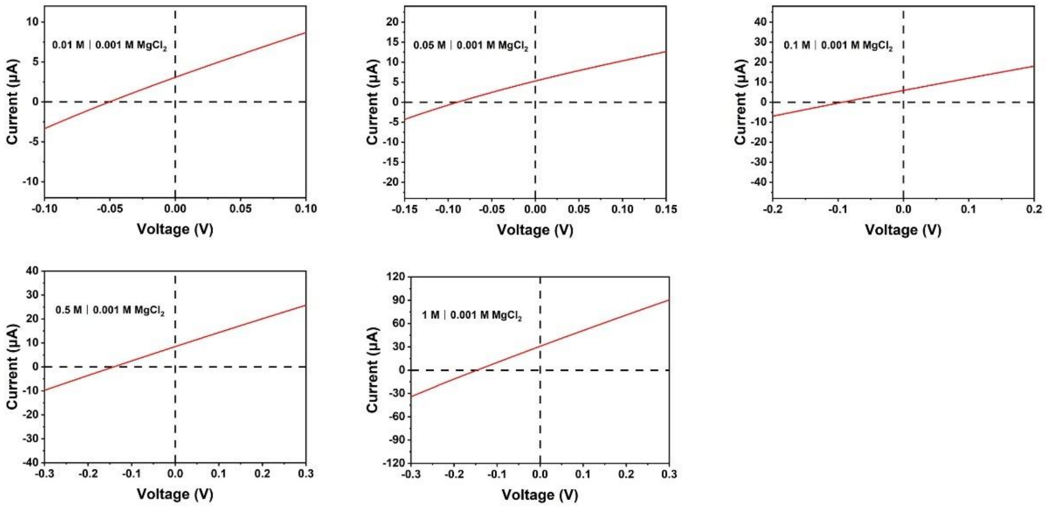

Fig. S36. ***IV*** **curves of NP-MXene membrane recorded in different $MgCl_2$ salinity gradient ratios of 10, 50, 100, 500 and 1000.**

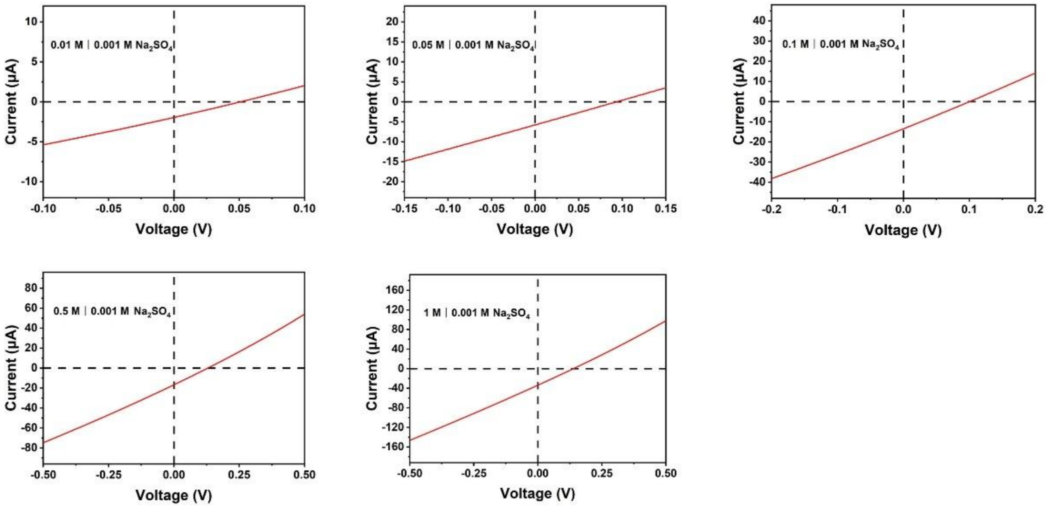

Fig. S37. ***IV*** **curves of NP-MXene membrane recorded in different Na_2SO_4 salinity gradient ratios of 10, 50, 100, 500 and 1000.**

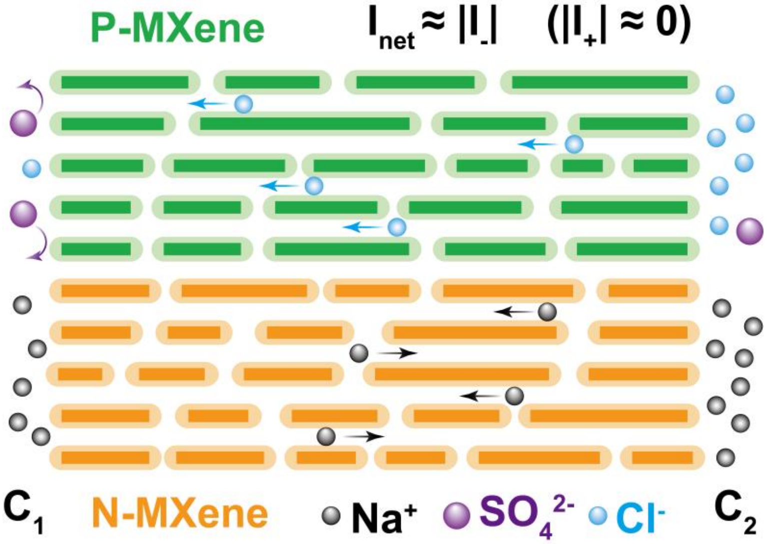

Fig. S38. Schematic of the ion diffusion in NP-MXene with the left side (C_1) being a high concentration of Na_2SO_4 and a low concentration of NaCl solution, and the right side (C_2) being a high concentration of NaCl and a low concentration of Na_2SO_4 solution.

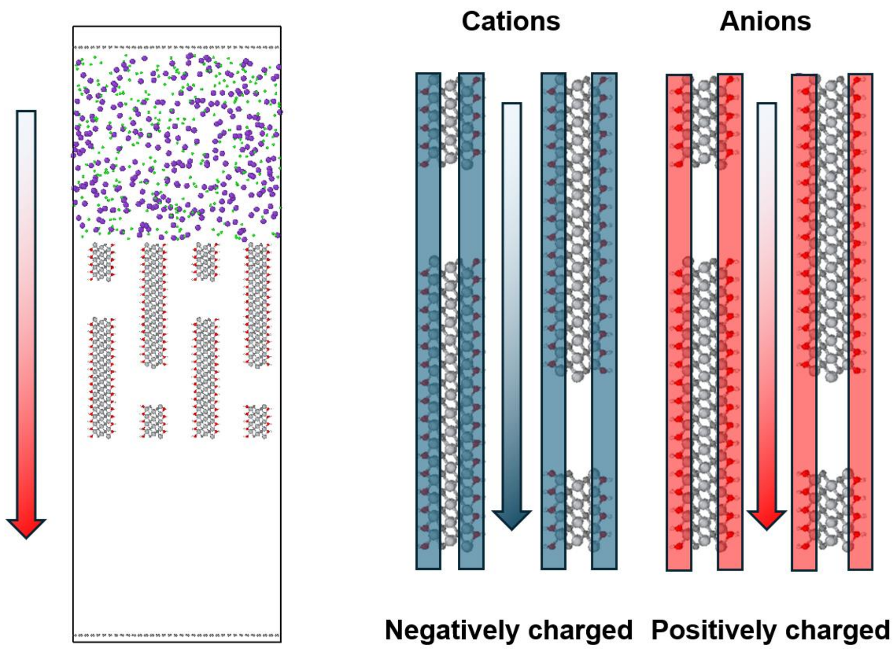

Fig. S39. Schematic diagram of NP-MXene membrane model. The actual width of the nanochannel is 10 Å, while the equilibrium width of the interaction is approximately 3.7 Å.

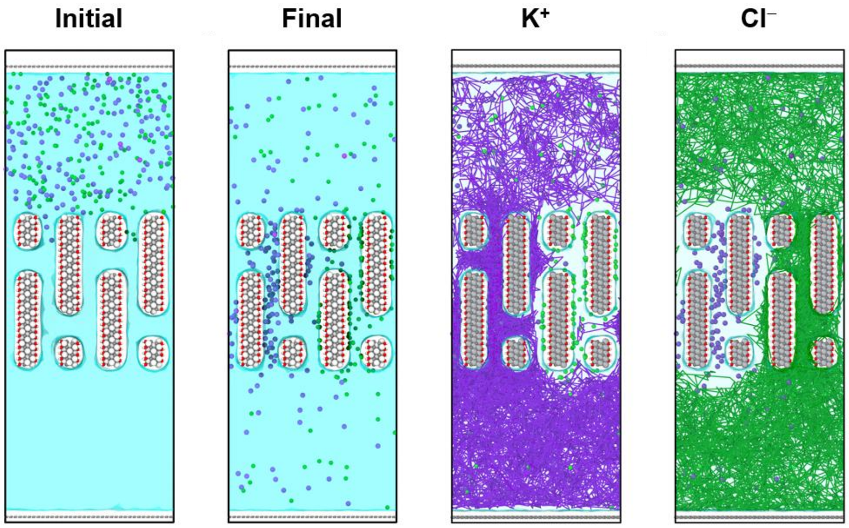

Fig. S40. MD simulation of ion transport across NP-MXene in KCl salinity gradient system. The initial state and final state of the NP-MXene membrane model in KCl salinity gradient system. Transport trajectories of K^+ and Cl^- in NP-MXene membrane model.

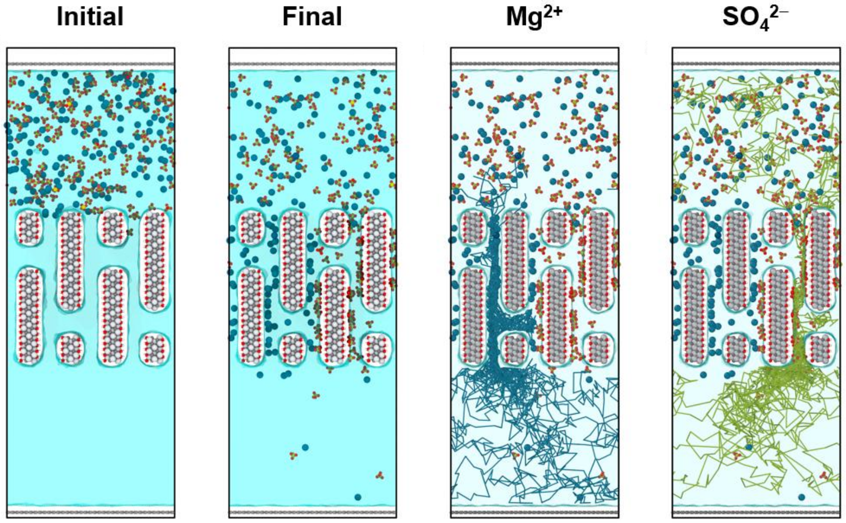

Fig. S41. MD simulation of ion transport across NP-MXene in $MgSO_4$ salinity gradient system. The initial state and final state of the NP-MXene membrane model in $MgSO_4$ salinity gradient system. Transport trajectories of Mg^{2+} and SO_4^{2-} in NP-MXene membrane model.

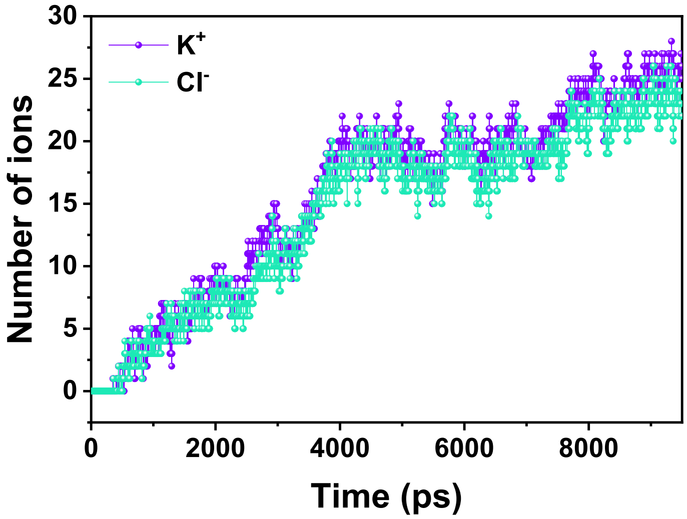

Fig. S42. The number of K^+ and Cl^- that pass through the NP-MXene membrane in KCl salinity gradient system.

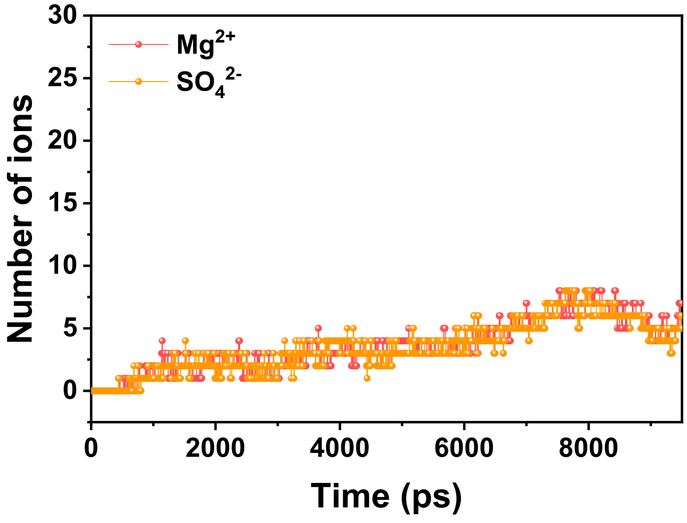

Fig. S43. The number of Mg^{2+} and SO_4^{2-} that pass through the NP-MXene membrane in $MgSO_4$ salinity gradient system.

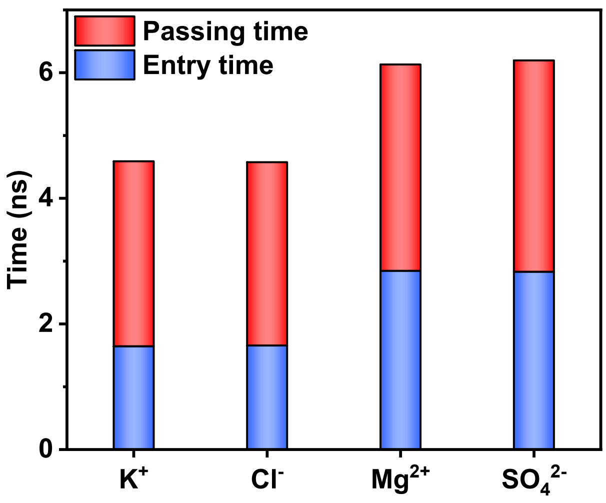

Fig. S44. Average entry time and passing time of K^+ and Cl^- (KCl salinity gradient system) and Mg^{2+} and SO_4^{2-} (Mg SO_4 salinity gradient system) passing the NP-MXene membrane.

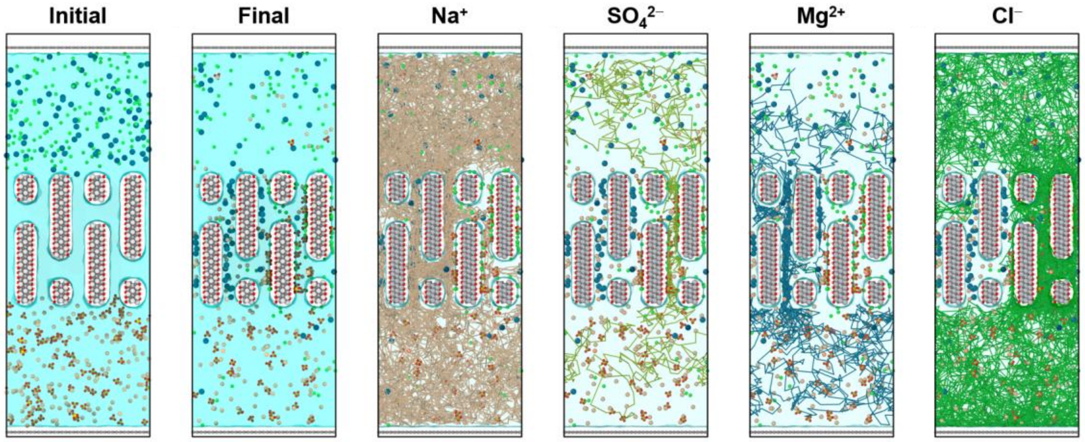

Fig. S45. MD simulation of ion transport across NP-MXene in Na_2SO_4 | $MgCl_2$ salinity gradient system. The initial state and final state of the NP-MXene membrane model in Na_2SO_4 | $MgCl_2$ salinity gradient system. Transport trajectories of Na^+, SO_4^{2-}, Mg^{2+} and Cl^- in NP-MXene membrane model.

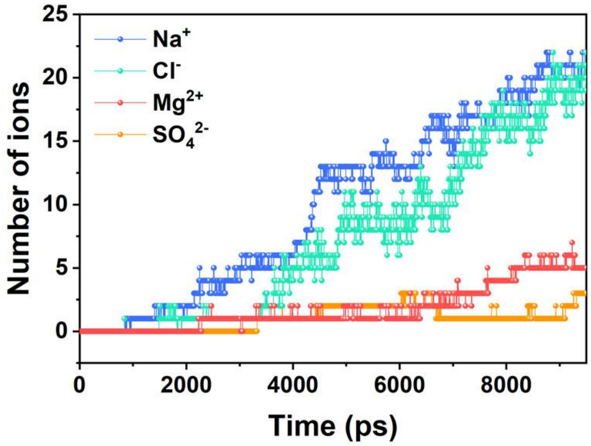

Fig. S46. The number of ions that pass through the NP-MXene membrane in Na_2SO_4 | $MgCl_2$ salinity gradient system.

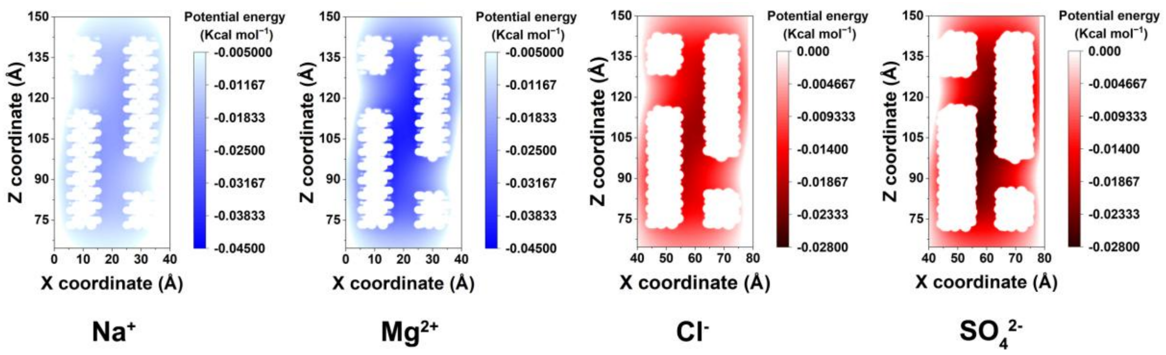

Fig. S47. The various ion migration energy barrier maps through NP-MXene channels in Na_2SO_4 | $MgCl_2$ salinity gradient system.

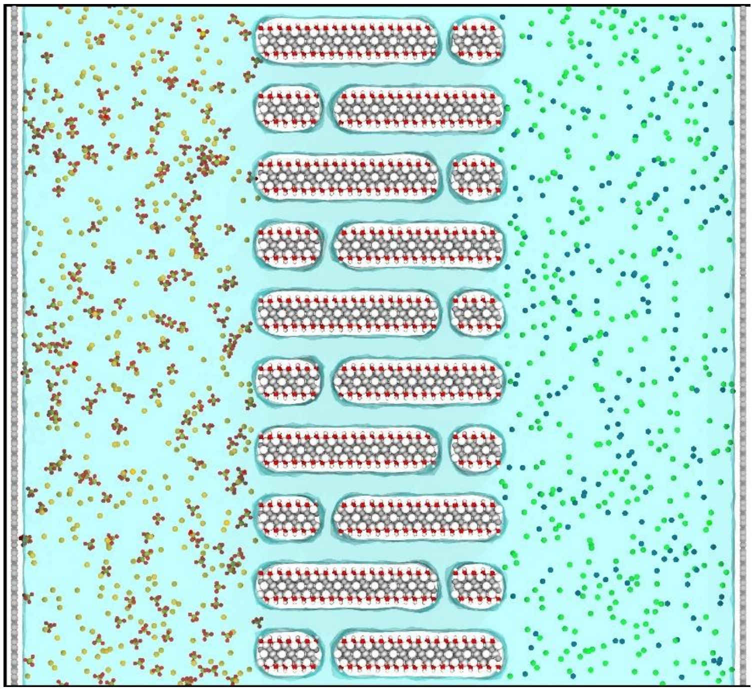

Fig. S48. Structural diagram of the MD simulation used to investigate ion transport trajectories in Na_2SO_4 | $MgCl_2$ salinity gradient system.

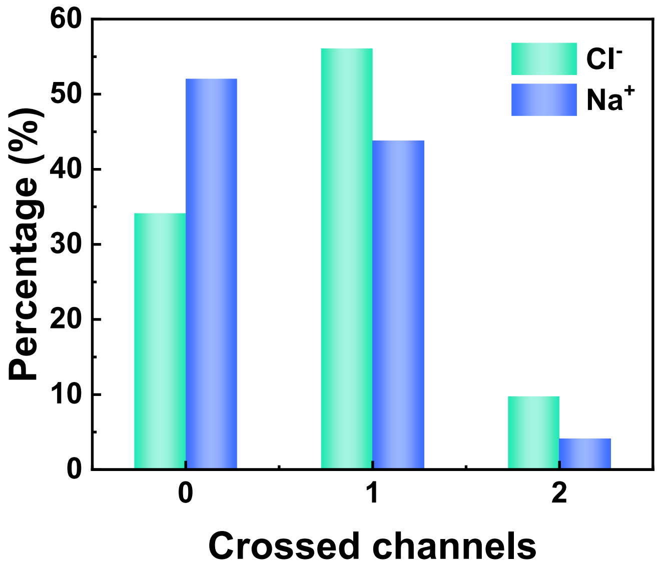

Fig. S49. Cross-channel transport of different ions during transport along the in-plane NP-MXene channels.

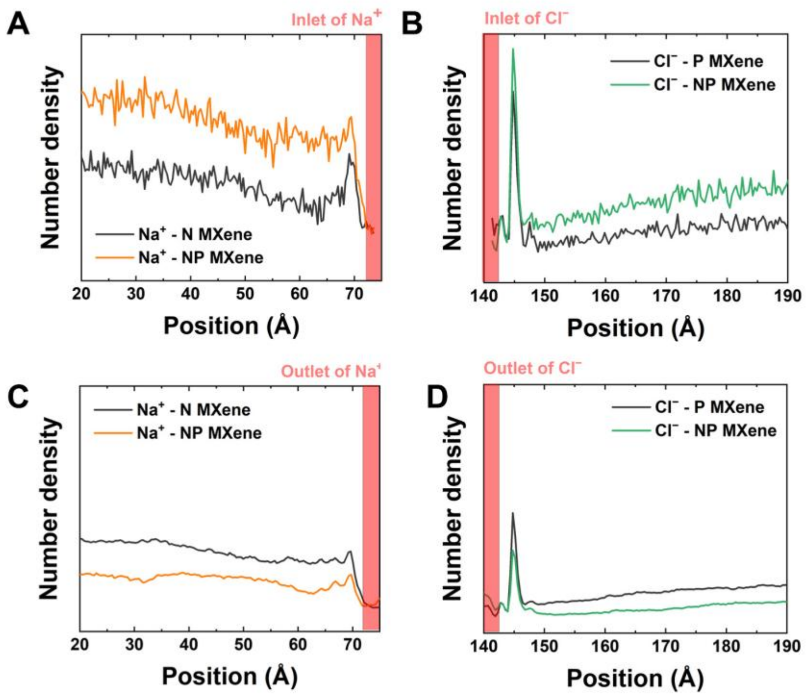

Fig. S50. MD simulation of ion concentration distributions at the inlet and outlet of NP MXene channels, and comparison with corresponding N-Mxene and P-MXene.

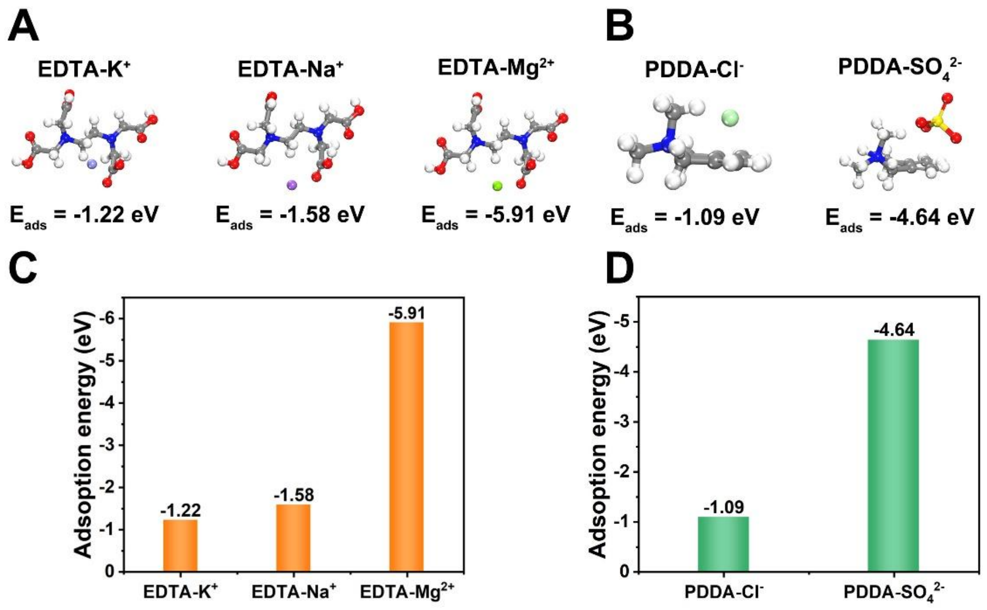

Fig. S51. The adsorption energy between an EDTA/PDDA molecules and various ions. (A) Configuration diagrams of cationic adsorption with EDTA molecule after structural optimization. **(B)** Configuration diagrams of anionic adsorption with PDDA molecule after structural optimization. **(C)** The adsorption energy between the EDTA molecule and the cations. **(D)** The adsorption energy between the PDDA molecule and the anions.

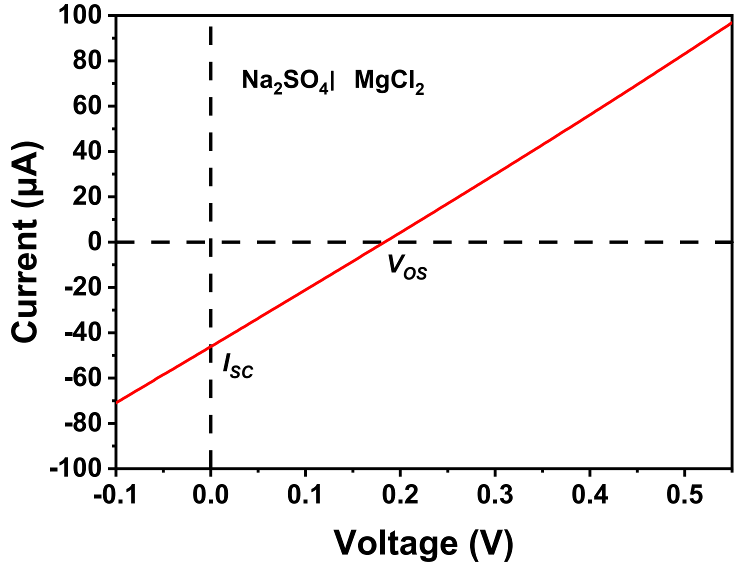

Fig. S52. ***IV*** **curves of NP-MXene membrane recorded in 50-fold Na_2SO_4 | $MgCl_2$ salinity gradient system.**

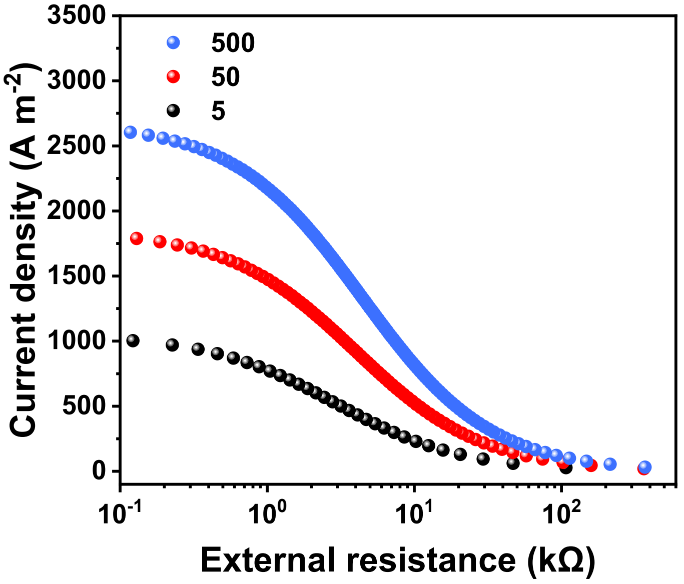

Fig. S53. The diffusion current density of a NP-MXene as a function of the increasing external resistance under three salinity gradients.

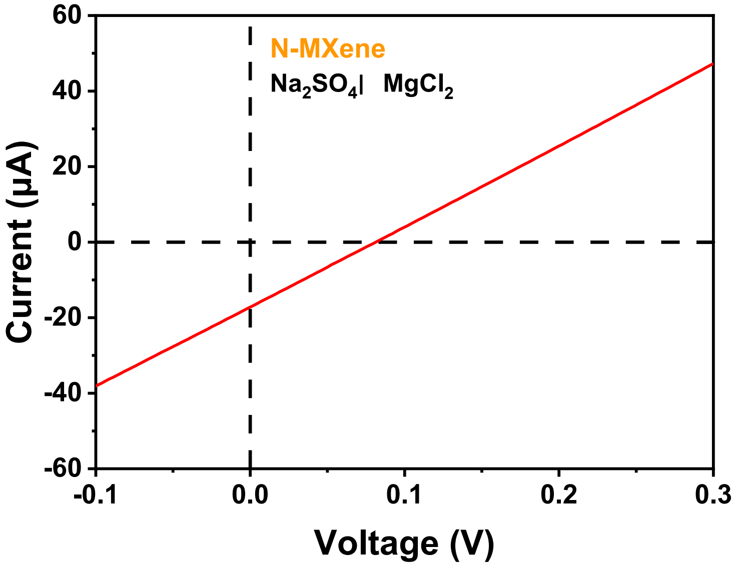

Fig. S54. *IV* curves of N-MXene membrane recorded in 50-fold Na_2SO_4 | $MgCl_2$ salinity gradient system.

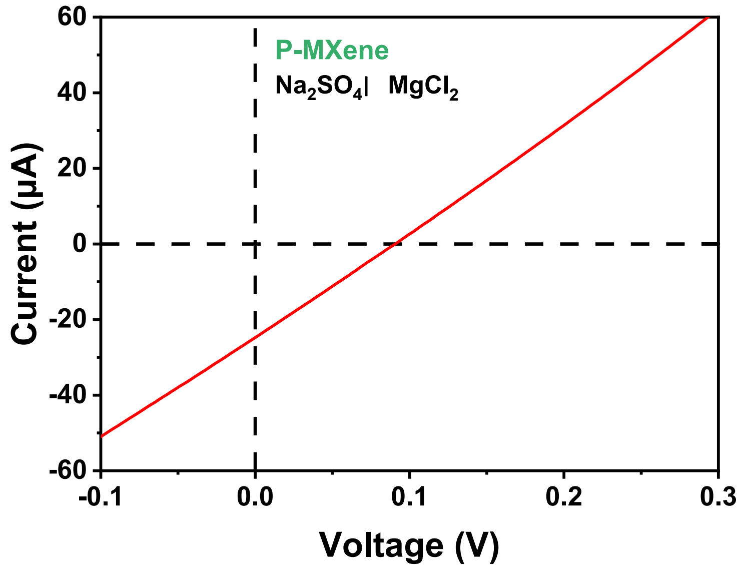

Fig. S55. *IV* curves of P-MXene membrane recorded in 50-fold Na_2SO_4 | $MgCl_2$ salinity gradient system.

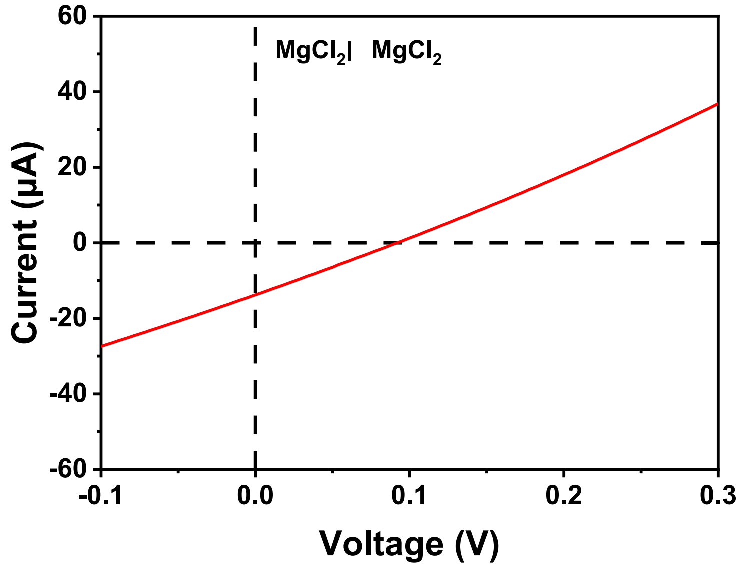

Fig. S56. *IV* curves of NP-MXene membrane recorded in 50-fold $MgCl_2$ | $MgCl_2$ salinity gradient system.

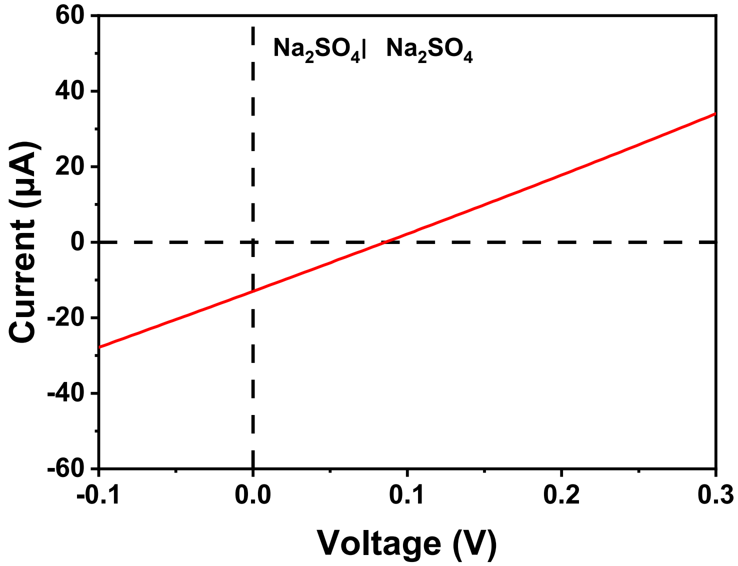

Fig. S57. ***IV*** **curves of NP-MXene membrane recorded in 50-fold Na_2SO_4 | Na_2SO_4 salinity gradient system.**

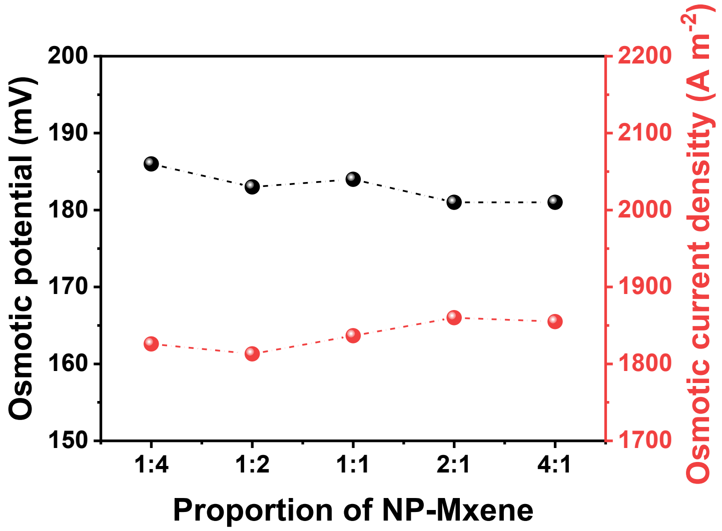

Fig. S58. Osmotic potential and osmotic current density as a function of NP-MXene proportion (N-MXene layer : P-MXene layer).

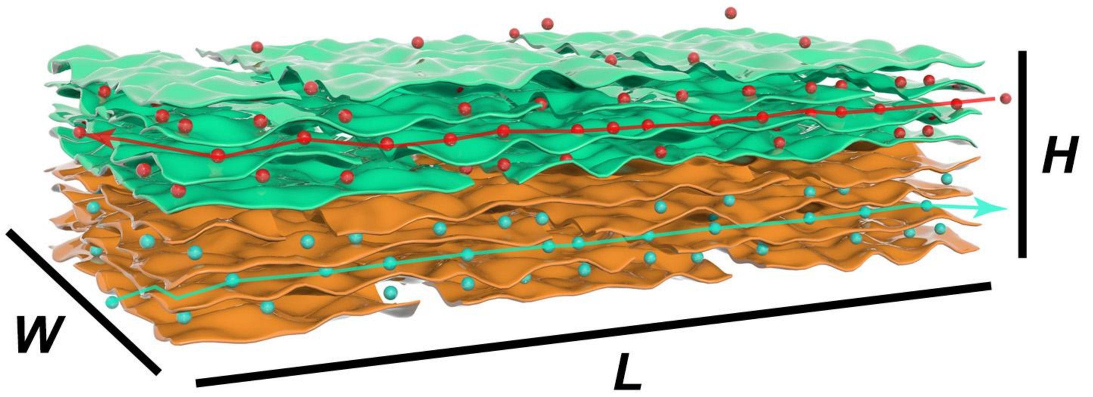

Fig. S59. Schematic of the length (*L*), width (*W*), and height (*H*) of the NP-MXene channels.

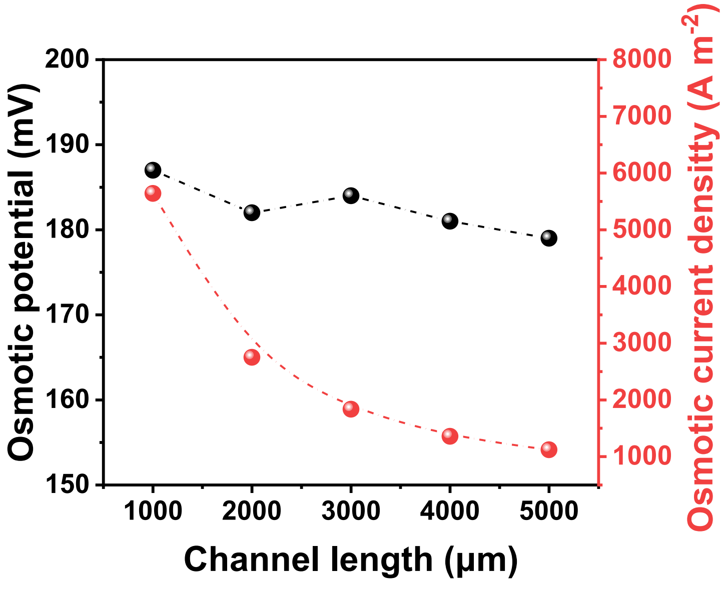

Fig. S60. Osmotic potential and osmotic current density as a function of channel length.

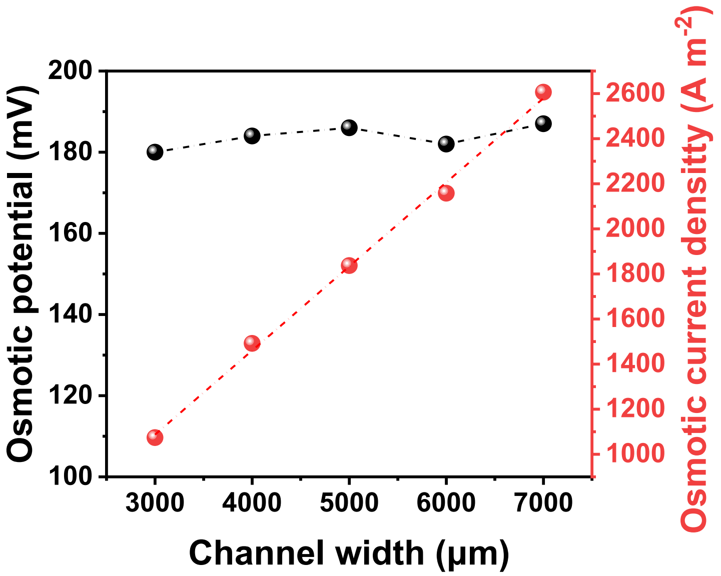

Fig. S61. Osmotic potential and osmotic current density as a function of channel width.

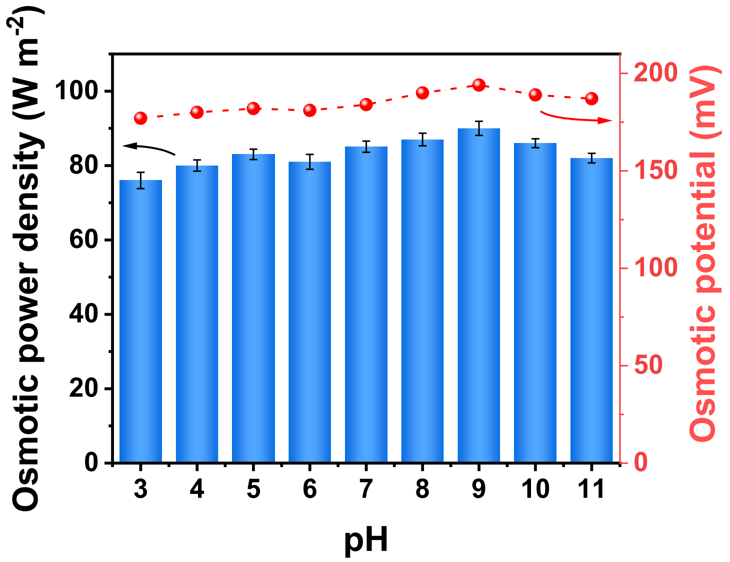

Fig. S62. Osmotic power density and osmotic potential of NP-MXene osmotic power sources as a function of pH.

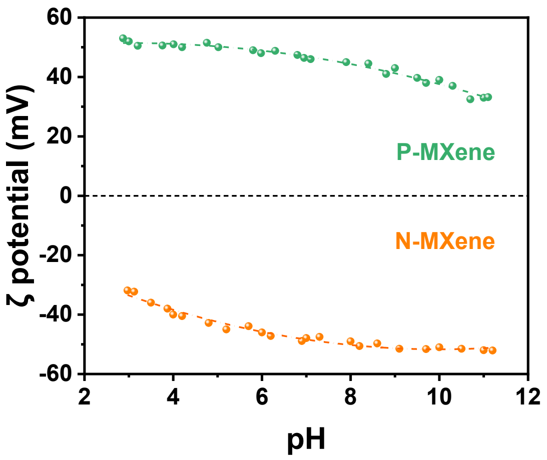

Fig. S63. Zeta potential of the N-MXene and P-MXene dispersions as a function of pH.

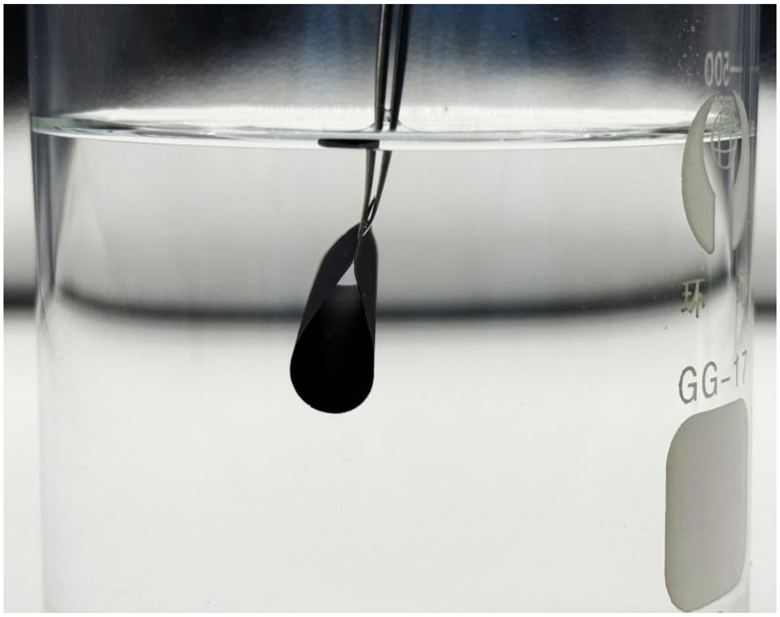

Fig. S64. Stability of the NP-MXene membrane in a mixed solution of 0.5 M Na_2SO_4 and 0.5 M $MgCl_2$.

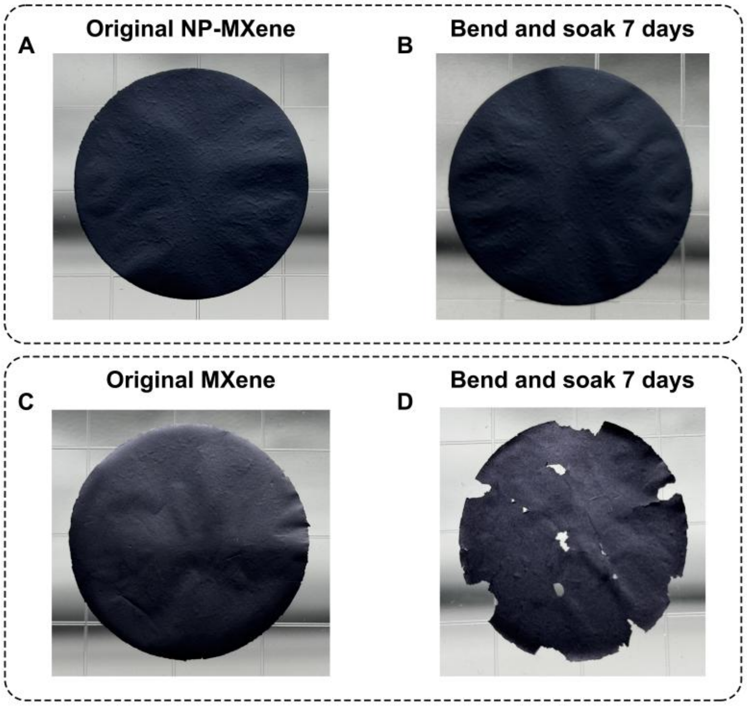

Fig. S65. Comparison of NP-MXene and MXene membranes after bending and soaking for 7 days in a mixed solution of Na_2SO_4 and $MgCl_2$. (A) Original NP-MXene membrane. (B) NP-MXene membrane after 7 days. (C) Original MXene membrane. (D) MXene membrane after 7 days.

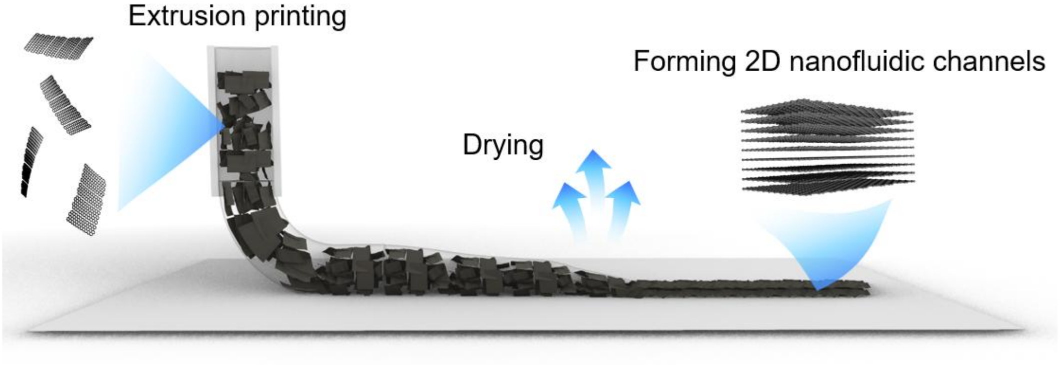

Fig. S66. Schematic diagram of the formation of 2D nanofluidic channels after extrusion printing and drying of the N-MXene and P-MXene aqueous inks. The inks preparation was referred to previous reports (*77*).

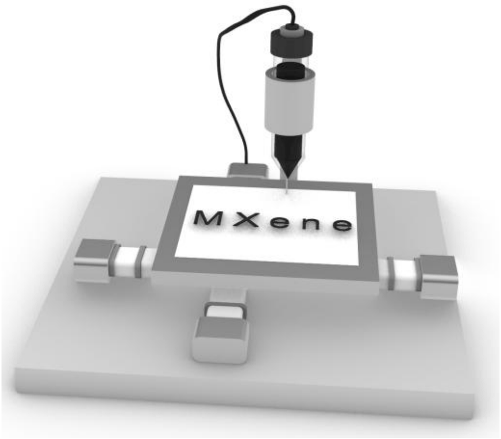

Fig. S67. Schematic illustration of the three-axis mechanical pneumatic liquid extrusion system for direct printing of MXene inks.

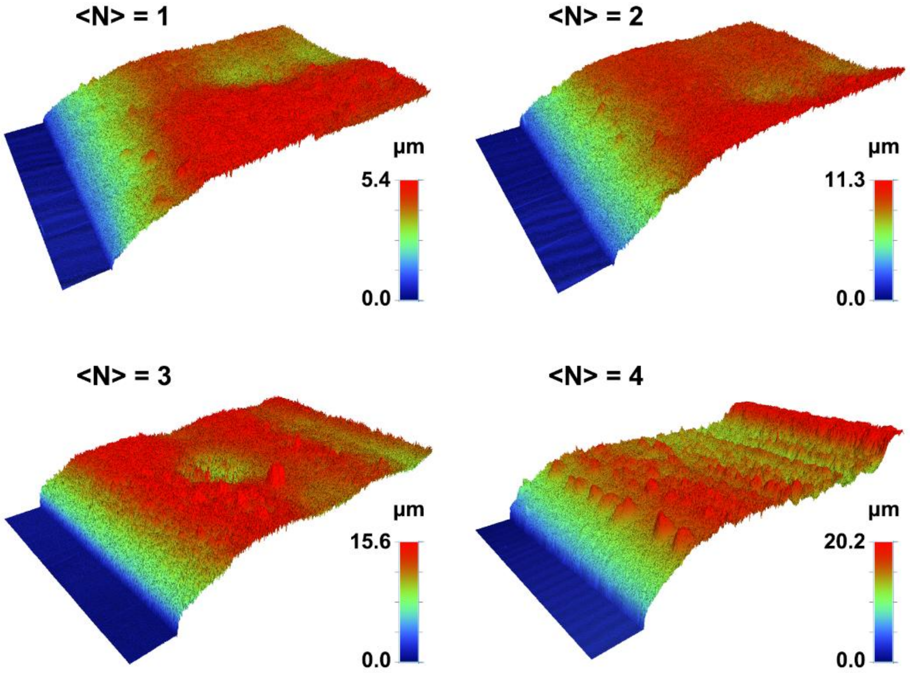

Fig. S68. The morphologies and heights of the printed NP-MXene channel membrane with different number of printing times <*N*> measured by an optical profilometer.

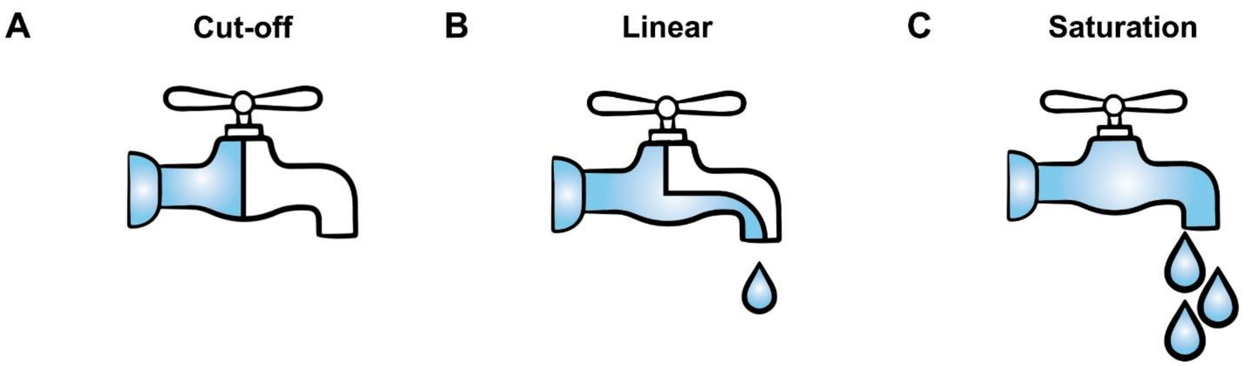

Fig. S69. Schematic of different states of the NP-MXene iontronic transistor. (A) "Cut-off" state. **(B)** "Linear" state. **(C)** "Saturation" state.

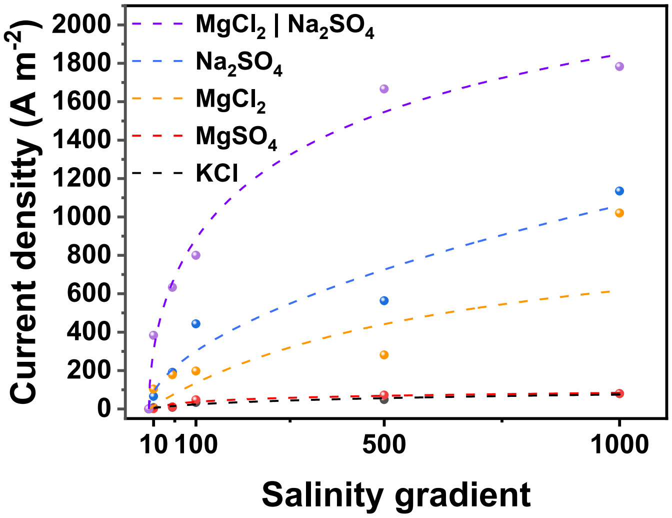

Fig. S70. Typical characteristic curves of NP-MXene iontronic transistor.

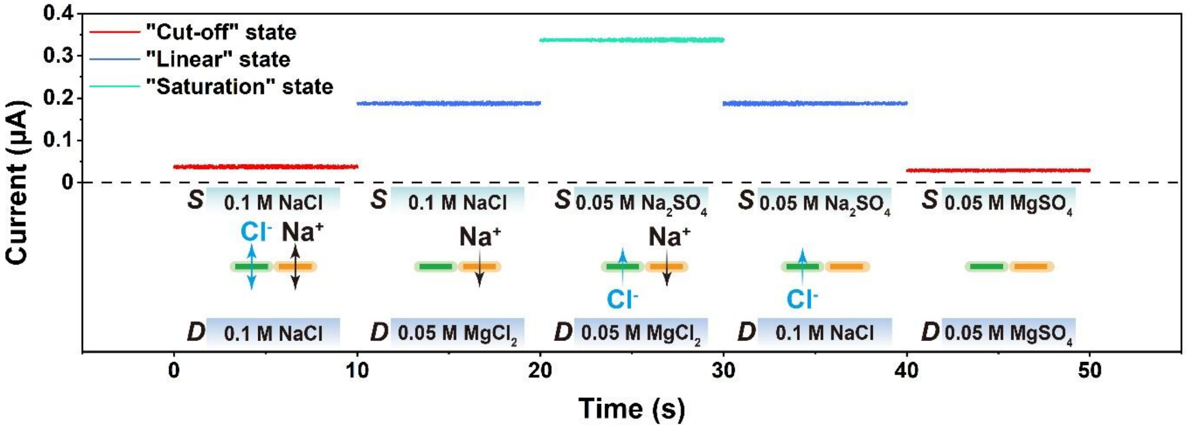

Fig. S71. Current signal switches when a stimulus is applied (ion) at lower concentration.

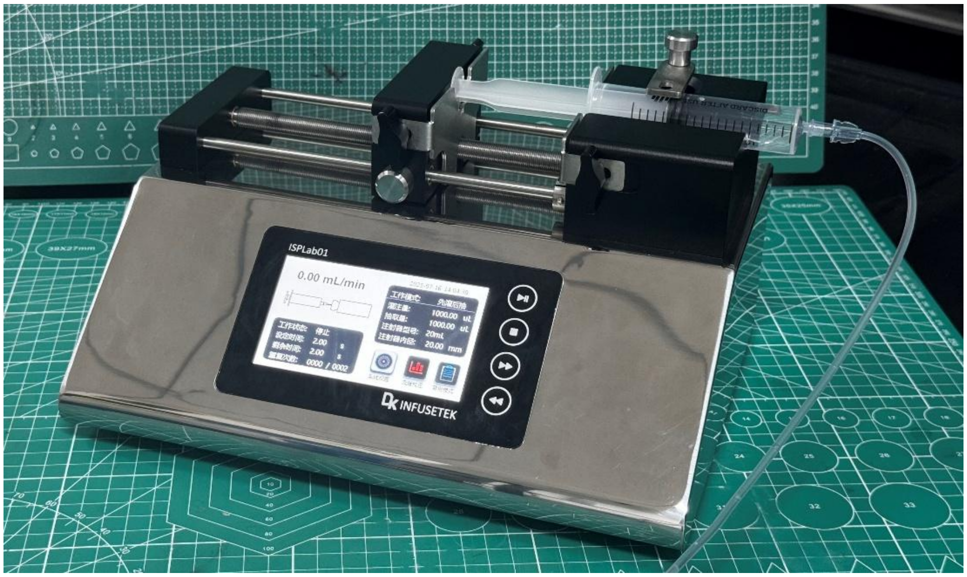

Fig. S72. The syringe pump to exchange solutions rapidly and precisely.

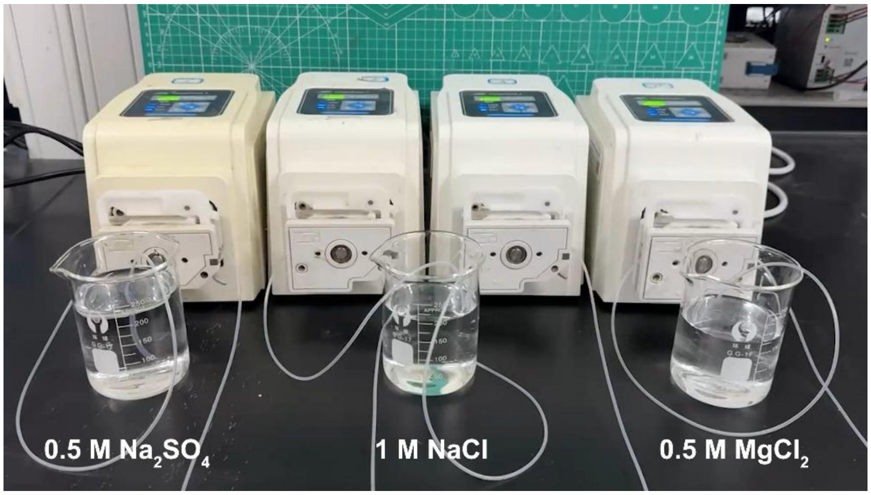

Fig. S73. The peristaltic pump to exchange solutions rapidly and precisely.

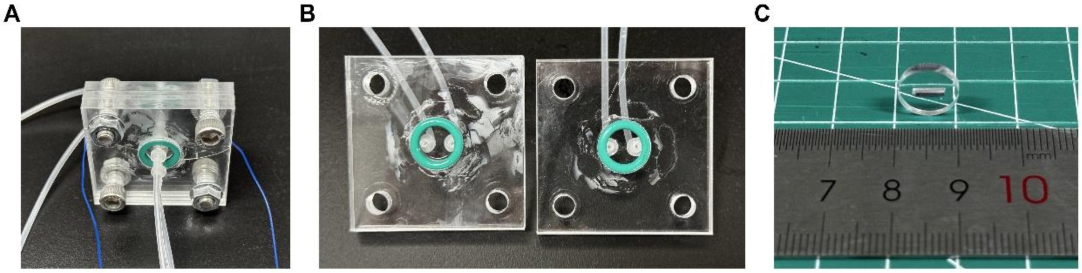

Fig. S74. The fully sealed fluidic system for precise information flow and iontronic human-machine interface interaction.

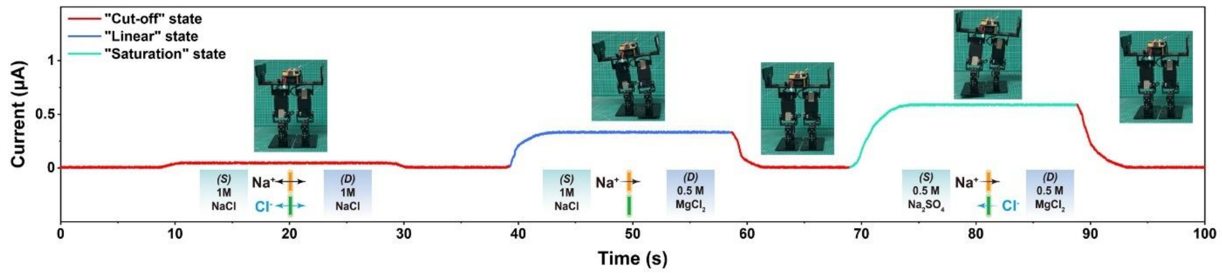

Fig. S75. The biomimetic Janus NP-MXene iontronic transistor for iontronic logic control.

01000001	A	01001000	H	01001111	O	01010110	V
01000010	B	01001001	I	01010000	P	01010111	W
01000011	C	01001010	J	01010001	Q		
01000100	D	01001011	K	01010010	R	01011000	X
01000101	E	01001100	L	01010011	S	01011001	Y
01000110	F	01001101	M	01010100	T		
01000111	G	01001110	N	01010101	U	01011010	Z

Fig. S76. The American Standard Code for Information Interchange (ASCII) is a widely used character encoding standard. For the 26 English letters, the first three bits of their binary representations are identical, meaning that only the last five bits are necessary for precise identification and transmission of the corresponding characters. This optimization reduces the amount of data required to convey textual information.

Supplementary Tables

Table S1. List of ions and their hydration energy.

Type of ions	Hydration energy (kJ mol^{-1})
Na^+	-365
K^+	-295
Mg^{2+}	-1830
Cl^-	-340
SO_4^{2-}	-1080

Data from reference (*55*).

Table S2. List of ions and their dehydrated bare radii and hydrated radii.

Type of ions	Bare radius (Å)	Hydrated radius (Å)
Na^+	0.95	3.58
K^+	1.33	3.31
Mg^{2+}	0.65	4.28
Cl^-	1.81	3.32
SO_4^{2-}	2.90	3.79

Data from reference (*52*).

Table S3. Summary of energy conversions in different membranes.

Membrane type	Condition	P_{max} (W m^{-2}) [a)]	V_{OS} (mV) [b)]	Efficiency (%)	Area (mm^2) [c)]	L (μm) [d)]	Ref
GOM	0.5 M | 0.01 M NaCl	0.77	140	36.6	0.2	10	(*57*)
PCGO	0.5 M | 0.01 M NaCl	5.26	96	41.4	0.075	3000	(*40*)
MoS_2	0.5 M | 0.01 M NaCl	5.2	64	32	0.04	4	(*78*)
MXene/BN	0.5 M | 0.01 M NaCl	2.3	72	21.1	0.0163	10	(*79*)
MXene/ANF	0.5 M | 0.01 M NaCl	3.7	87	20	0.03	4.5	(*42*)
MXene	0.5 M | 0.01 M NaCl	4.6	94	44.3	0.2	10	(*80*)
Silk fibroin	0.5 M | 0.01 M NaCl	4.06	65	4.3	0.03	0.1	(*81*)
PES-Py/HS20	0.5 M | 0.01 M NaCl	2.66	85	35.7	-	11	(*82*)
Polymer/MOF	0.5 M | 0.01 M KCl	2.87	78	29.7	0.03	85	(*83*)
MS/AAO	0.5 M | 0.01 M NaCl	4.5	36	3.5	0.03	60.1	(*84*)
IDM	0.5 M | 0.01 M NaCl	3.46	87	37.3	0.03	64.2	(*85*)

BHMXene	0.5 M | 0.01 M NaCl	8.6	89	39.2	0.03	4	(*56*)
$COF\text{-}SO_3H$	0.5 M | 0.01 M NaCl	43.2	57	16	0.03	0.1	(*86*)
ZnTPP-COF	0.5 M | 0.01 M NaCl	135.8	28	3.89	3.14×10^{-6}	0.0011	(*41*)
MXene fibers	0.5 M | 0.01 M NaCl	12.3	96	45.7	0.0012	1000	(*87*)
**This work**	**0.5 M Na_2SO_4 (0.01 M $MgCl_2$) | 0.5 M $MgCl_2$ (0.01 M Na_2SO_4)**	**85.08**	**184**	**-**	**0.025**	**3000**	-

[a] The maximum osmotic power density (P_{max})
[b] The osmotic potential (V_{OS})
[c] Area ($S = W * H$)
[d] Channel length (L)
[*] See fig. S59 for details.

Table S4. Summary of state-of-the-art osmotic power density of large-area membranes.

Membrane type	Power density (W m^{-2})	Device area (mm^2)	Ref
MXene/Kevlar nanofiber composite	4.10	0.03	(*42*)
Polyelectrolyte hydrogel/aramid nanofiber heterogeneous	4.97	0.03	(*88*)
Mushroom-shaped nanochannel array	14.6	0.008	(*89*)
UiO-66-NH_2 MOF	11	0.03	(*12*)
ZnTPP-COF	14.63	0.25	(*41*)
$COF-SO_3H$	43.2	0.03	(*90*)
**NP-MXene**	**40.32**	**0.30**	**This work**

Supplementary Movies

Movie S1.

The biomimetic Janus NP-MXene iontronic transistor for iontronic logic control

REFERENCES AND NOTES

1. F. Yang, P. Peng, Z.-Y. Yan, H. Fan, X. Li, S. Li, H. Liu, T.-L. Ren, Y. Zhou, Z. L. Wang, D. Wei, Vertical iontronic energy storage based on osmotic effects and electrode redox reactions. *Nat. Energy* **9**, 263–271 (2024).

2. Y. Hou, X. Hou, Bioinspired nanofluidic iontronics. *Science* **373**, 628–629 (2021).

3. L. Cao, I.-C. Chen, Z. Li, X. Liu, M. Mubashir, R. A. Nuaimi, Z. Lai, Switchable Na^+ and K^+ selectivity in an amino acid functionalized 2D covalent organic framework membrane. *Nat. Commun.* **13**, 7894 (2022).

4. H. Qian, D. Wei, Z. L. Wang, Bionic iontronics based on nano-confined structures. *Nano Res* **16**, 11718–11730 (2023).

5. P. Peng, H. Qian, J. Liu, Z. Wang, D. Wei, Bioinspired ionic control for energy and information flow. *Int. J. Smart Nano Mater.* **15**, 198–221 (2024).

6. P. Peng, F. Yang, X. Li, S. Li, Z. Wang, D. Wei, High-power iontronics enabled by nanoconfined ion dynamics. *Cell Rep. Phys. Sci.* **5**, 101824 (2024).

7. J. Shen, G. Liu, Y. Han, W. Jin, Artificial channels for confined mass transport at the sub-nanometre scale. *Nat. Rev. Mater.* **6**, 294–312 (2021).

8. M. K. F. Wikstrom, Proton pump coupled to cytochrome c oxidase in mitochondria. *Nature* **266**, 271–273 (1977).

9. B. Chance, L. Mela, Proton movements in mitochondrial membranes. *Nature* **212**, 372–376 (1966).

10. J. F. Allen, Photosynthesis of ATP—Electrons, proton pumps, rotors, and poise. *Cell* **110**, 273–276 (2002).

11. Y. Teng, P. Liu, L. Fu, X.-Y. Kong, L. Jiang, L. Wen, Bioinspired nervous signal transmission system based on two-dimensional laminar nanofluidics: From electronics to ionics. *Proc. Natl. Acad. Sci. U.S.A.* **117**, 16743–16748 (2020).

12. Y.-C. Liu, L.-H. Yeh, M.-J. Zheng, K. C.-W. Wu, Highly selective and high-performance osmotic power generators in subnanochannel membranes enabled by metal-organic frameworks. *Sci. Adv.* **7**, eabe9924 (2021).

13. K. Xiao, L. Jiang, M. Antonietti, Ion transport in nanofluidic devices for energy harvesting. *Joule* **3**, 2364–2380 (2019).

14. H. Qin, H. Wu, S.-M. Zeng, F. Yi, S.-Y. Qin, Y. Sun, L. Ding, H. Wang, Harvesting osmotic energy from proton gradients enabled by two-dimensional $Ti_3C_2T_x$ MXene membranes. *Adv. Membr.* **2**, 100046 (2022).

15. J. Feng, K. Liu, M. Graf, D. Dumcenco, A. Kis, M. Di Ventra, A. Radenovic, Observation of ionic Coulomb blockade in nanopores. *Nat. Mater.* **15**, 850–855 (2016).

16. N. Kavokine, S. Marbach, A. Siria, L. Bocquet, Ionic Coulomb blockade as a fractional Wien effect. *Nat. Nanotechnol.* **14**, 573–578 (2019).

17. H. Zhan, Z. Xiong, C. Cheng, Q. Liang, J. Z. Liu, D. Li, Solvation-involved nanoionics: New opportunities from 2D nanomaterial laminar membranes. *Adv. Mater.* **32**, 1904562 (2020).

18. S. Kondrat, A. Kornyshev, Superionic state in double-layer capacitors with nanoporous electrodes. *J. Phys. Condens. Matter* **23**, 022201 (2011).

19. X. Li, R. Li, S. Li, Z. L. Wang, D. Wei, Triboiontronics with temporal control of electrical double layer formation. *Nat. Commun.* **15**, 6182 (2024).

20. D. Wei, Writable electrochemical energy source based on graphene oxide. *Sci. Rep.* **5**, 15173 (2015).

21. D. Wei, F. Yang, Z. Jiang, Z. Wang, Flexible iontronics based on 2D nanofluidic material. *Nat. Commun.* **13**, 4965 (2022).

22. L. Yang, L. N. Y. Cao, S. Li, P. Peng, H. Qian, G. Amaratunga, F. Yang, Z. L. Wang, D. Wei, MOFs/MXene nano-hierarchical porous structures for efficient ion dynamics. *Nano Energy* **129**, 110076 (2024).

23. L. Yang, S. Li, H. Qian, Z. Wang, Z. L. Wang, D. Wei, Osmotic power generation based on nanoconfined materials. *MRS Energy Sustain.* **11**, 193–218 (2024).

24. M. Feng, S. Feng, T. Yu, S. Zhu, H. Cai, X. He, Y. Liu, M. He, X. Bu, J. Huang, Y. Zhou, Versatile and comfortable janus fabrics for switchable personal thermal management and electromagnetic interference shielding. *Adv. Fiber Mater.* **6**, 911–924 (2024).

25. M. Feng, H. Cai, S. Feng, Y. Liu, Z. Li, X. He, S. Liang, X. Bu, J. Huang, Y. Zhou, Asymmetric gradient porous fabric with dynamically tunable thermal management and electromagnetic interference shielding via delayed phase separation. *Adv. Funct. Mater.* **35**, 2422487 (2025).

26. K. Xiao, L. Chen, R. Chen, T. Heil, S. D. C. Lemus, F. Fan, L. Wen, L. Jiang, M. Antonietti, Artificial light-driven ion pump for photoelectric energy conversion. *Nat. Commun.* **10**, 74 (2019).

27. L. Yang, F. Yang, X. Liu, K. Li, Y. Zhou, Y. Wang, T. Yu, M. Zhong, X. Xu, L. Zhang, W. Shen, D. Wei, A moisture-enabled fully printable power source inspired by electric eels. *Proc. Natl. Acad. Sci. U.S.A.* **118**, e2023164118 (2021).

28. P. Peng, P. Shen, H. Qian, J. Liu, H. Lu, Y. Jiao, F. Yang, H. Liu, T. Ren, Z. Wang, D. Wei, Photochemical iontronics with multitype ionic signal transmission at single pixel for self-driven color and tridimensional vision. *Device* **3**, 100574 (2025).

29. P. Cao, Y. Wang, L. Yu, M. Wang, L. Zhao, X. Hou, Dynamic asymmetric mechanical responsive carbon nanotube fiber for ionic logic gate. *Chin. Chem. Lett.* **35**, 109421 (2024).

30. W. Liu, T. Mei, Z. Cao, C. Li, Y. Wu, L. Wang, G. Xu, Y. Chen, Y. Zhou, S. Wang, Y. Xue, Y. Yu, X.-Y. Kong, R. Chen, B. Tu, K. Xiao, Bioinspired carbon nanotube–Based nanofluidic

ionic transistor with ultrahigh switching capabilities for logic circuits. *Sci. Adv.* **10**, eadj7867 (2024).

31. T. Xiong, C. Li, X. He, B. Xie, J. Zong, Y. Jiang, W. Ma, F. Wu, J. Fei, P. Yu, L. Mao, Neuromorphic functions with a polyelectrolyte-confined fluidic memristor. *Science* **379**, 156–161 (2023).

32. Q. Li, K. Zhou, B. Zhu, X. Liu, J. Lao, J. Gao, L. Jiang, Artificial sodium channels for enhanced osmotic energy harvesting. *J. Am. Chem. Soc.* **145**, 28038–28048 (2023).

33. A. R. Koltonow, J. Huang, Two-dimensional nanofluidics. *Science* **351**, 1395–1396 (2016).

34. H. Qian, P. Peng, H. Fan, Z. Yang, L. Yang, Y. Zhou, D. Tan, F. Yang, M. Willatzen, G. Amaratunga, Z. Wang, D. Wei, Horizontal transport in $Ti_3C_2T_x$ MXene for highly efficient osmotic energy conversion from Saline-Alkali environments. *Angew. Chem. Int. Ed. Engl.* **63**, e202414984 (2024).

35. R. Xu, Y. Kang, W. Zhang, B. Pan, X. Zhang, Two-dimensional MXene membranes with biomimetic sub-nanochannels for enhanced cation sieving. *Nat. Commun.* **14**, 4907 (2023).

36. Z.-K. Li, Y. Wei, X. Gao, L. Ding, Z. Lu, J. Deng, X. Yang, J. Caro, H. Wang, Antibiotics separation with MXene membranes based on regularly stacked high-aspect-ratio nanosheets. *Angew. Chem. Int. Ed.* **59**, 9751–9756 (2020).

37. J. Wang, Z. Zhang, J. Zhu, M. Tian, S. Zheng, F. Wang, X. Wang, L. Wang, Ion sieving by a two-dimensional $Ti_3C_2T_x$ alginate lamellar membrane with stable interlayer spacing. *Nat. Commun.* **11**, 3540 (2020).

38. S. Wan, X. Li, Y. Wang, Y. Chen, X. Xie, R. Yang, A. P. Tomsia, L. Jiang, Q. Cheng, Strong sequentially bridged MXene sheets. *Proc. Natl. Acad. Sci. U.S.A.* **117**, 27154–27161 (2020).

39. G. Liu, S. Liu, K. Ma, H. Wang, X. Wang, G. Liu, W. Jin, Polyelectrolyte functionalized Ti2CTx MXene Membranes for pervaporation dehydration of isopropanol/water mixtures. *Ind. Eng. Chem. Res.* **59**, 4732–4741 (2020).

40. S. Kim, S. Choi, H. G. Lee, D. Jin, G. Kim, T. Kim, J. S. Lee, W. Shim, Neuromorphic van der Waals crystals for substantial energy generation. *Nat. Commun.* **12**, 47 (2021).

41. J. Yang, B. Tu, G. Zhang, P. Liu, K. Hu, J. Wang, Z. Yan, Z. Huang, M. Fang, J. Hou, Q. Fang, X. Qiu, L. Li, Z. Tang, Advancing osmotic power generation by covalent organic framework monolayer. *Nat. Nanotechnol.* **17**, 622–628 (2022).

42. Z. Zhang, S. Yang, P. Zhang, J. Zhang, G. Chen, X. Feng, Mechanically strong MXene/Kevlar nanofiber composite membranes as high-performance nanofluidic osmotic power generators. *Nat. Commun.* **10**, 2920 (2019).

43. W. Guo, L. Cao, J. Xia, F.-Q. Nie, W. Ma, J. Xue, Y. Song, D. Zhu, Y. Wang, L. Jiang, Energy harvesting with single-ion-selective nanopores: A concentration-gradient-driven nanofluidic power source. *Adv. Funct. Mater.* **20**, 1339–1344 (2010).

44. C. Duan, A. Majumdar, Anomalous ion transport in 2-nm hydrophilic nanochannels. *Nat. Nanotechnol.* **5**, 848–852 (2010).

45. Z. Siwy, E. Heins, C. C. Harrell, P. Kohli, C. R. Martin, Conical-nanotube ion-current rectifiers: The role of surface charge. *J. Am. Chem. Soc.* **126**, 10850–10851 (2004).

46. J. Feng, M. Graf, K. Liu, D. Ovchinnikov, D. Dumcenco, M. Heiranian, V. Nandigana, N. R. Aluru, A. Kis, A. Radenovic, Single-layer MoS_2 nanopores as nanopower generators. *Nature* **536**, 197–200 (2016).

47. J. Lu, H. Zhang, J. Hou, X. Li, X. Hu, Y. Hu, C. D. Easton, Q. Li, C. Sun, A. W. Thornton, M. R. Hill, X. Zhang, G. Jiang, J. Z. Liu, A. J. Hill, B. D. Freeman, L. Jiang, H. Wang, Efficient metal ion sieving in rectifying subnanochannels enabled by metal–Organic frameworks. *Nat. Mater.* **19**, 767–774 (2020).

48. A. Esfandiar, B. Radha, F. C. Wang, Q. Yang, S. Hu, S. Garaj, R. R. Nair, A. K. Geim, K. Gopinadhan, Size effect in ion transport through angstrom-scale slits. *Science* **358**, 511–513 (2017).

49. H. Zhang, J. Hou, Y. Hu, P. Wang, R. Ou, L. Jiang, J. Z. Liu, B. D. Freeman, A. J. Hill, H. Wang, Ultrafast selective transport of alkali metal ions in metal organic frameworks with subnanometer pores. *Sci. Adv.* **4**, eaaq0066 (2018).

50. P. Wang, M. Wang, F. Liu, S. Ding, X. Wang, G. Du, J. Liu, P. Apel, P. Kluth, C. Trautmann, Y. Wang, Ultrafast ion sieving using nanoporous polymeric membranes. *Nat. Commun.* **9**, 569 (2018).

51. X. Li, H. Zhang, P. Wang, J. Hou, J. Lu, C. D. Easton, X. Zhang, M. R. Hill, A. W. Thornton, J. Z. Liu, B. D. Freeman, A. J. Hill, L. Jiang, H. Wang, Fast and selective fluoride ion conduction in sub-1-nanometer metal-organic framework channels. *Nat. Commun.* **10**, 2490 (2019).

52. E. R. Nightingale Jr., Phenomenological theory of ion solvation. Effective Radii of hydrated ions. *J. Phys. Chem.* **63**, 1381–1387 (1959).

53. Y. Xue, Y. Xia, S. Yang, Y. Alsaid, K. Y. Fong, Y. Wang, X. Zhang, Atomic-scale ion transistor with ultrahigh diffusivity. *Science* **372**, 501–503 (2021).

54. J. Abraham, K. S. Vasu, C. D. Williams, K. Gopinadhan, Y. Su, C. T. Cherian, J. Dix, E. Prestat, S. J. Haigh, I. V. Grigorieva, P. Carbone, A. K. Geim, R. R. Nair, Tunable sieving of ions using graphene oxide membranes. *Nat. Nanotechnol.* **12**, 546–550 (2017).

55. Y. Marcus, A simple empirical model describing the thermodynamics of hydration of ions of widely varying charges, sizes, and shapes. *Biophys. Chem.* **51**, 111–127 (1994).

56. L. Ding, M. Zheng, D. Xiao, Z. Zhao, J. Xue, S. Zhang, J. Caro, H. Wang, Bioinspired $Ti_3C_2T_x$ MXene-based ionic diode membrane for high-efficient osmotic energy conversion. *Angew. Chem. Int. Ed.* **61**, e202206152 (2022).

57. J. Ji, Q. Kang, Y. Zhou, Y. Feng, X. Chen, J. Yuan, W. Guo, Y. Wei, L. Jiang, Osmotic power generation with positively and negatively charged 2D nanofluidic membrane pairs. *Adv. Funct. Mater.* **27**, 1603623 (2017).

58. L. Cao, F. Xiao, Y. Feng, W. Zhu, W. Geng, J. Yang, X. Zhang, N. Li, W. Guo, L. Jiang, Anomalous channel-length dependence in nanofluidic osmotic energy conversion. *Adv. Funct. Mater.* **27**, 1604302 (2017).

59. F. J. Sigworth, Life's transistors. *Nature* **423**, 21–22 (2003).

60. T. Mei, W. Liu, G. Xu, Y. Chen, M. Wu, L. Wang, K. Xiao, Ionic transistors. *ACS Nano* **18**, 4624–4650 (2024).

61. J.-P. Colinge, C.-W. Lee, A. Afzalian, N. D. Akhavan, R. Yan, I. Ferain, P. Razavi, B. O'Neill, A. Blake, M. White, A.-M. Kelleher, B. McCarthy, R. Murphy, Nanowire transistors without junctions. *Nat. Nanotechnol.* **5**, 225–229 (2010).

62. K. Xiao, C. Wan, L. Jiang, X. Chen, M. Antonietti, Bioinspired ionic sensory systems: The successor of electronics. *Adv. Mater.* **32**, e2000218 (2020).

63. R. Sarpeshkar, Analog versus digital: Extrapolating from electronics to neurobiology. *Neural Comput.* **10**, 1601–1638 (1998).

64. T. Mei, W. Liu, F. Sun, Y. Chen, G. Xu, Z. Huang, Y. Jiang, S. Wang, L. Chen, J. Liu, F. Fan, K. Xiao, Bio-inspired two-dimensional nanofluidic ionic transistor for neuromorphic signal processing. *Angew. Chem. Int. Ed. Engl.* **63**, e202401477 (2024).

65. S. Plimpton, Fast parallel algorithms for short-range molecular dynamics. *J. Comput. Phys.* **117**, 1–19 (1995).

66. J. L. F. Abascal, C. Vega, A general purpose model for the condensed phases of water: TIP4P/2005. *J. Chem. Phys.* **123**, 234505 (2005).

67. A. K. Rappe, C. J. Casewit, K. S. Colwell, W. A. Goddard III, W. M. Skiff, UFF, a full periodic table force field for molecular mechanics and molecular dynamics simulations. *J. Am. Chem. Soc.* **114**, 10024–10035 (1992).

68. I. M. Zeron, J. L. F. Abascal, C. Vega, A force field of Li^+, Na^+, K^+, Mg^{2+}, Ca^{2+}, Cl^-, and SO_4^{2-} in aqueous solution based on the TIP4P/2005 water model and scaled charges for the ions. *J. Chem. Phys.* **151**, 134504 (2019).

69. J. Halim, K. M. Cook, M. Naguib, P. Eklund, Y. Gogotsi, J. Rosen, M. W. Barsoum, X-ray photoelectron spectroscopy of select multi-layered transition metal carbides (MXenes). *Appl. Surf. Sci.* **362**, 406–417 (2016).

70. L. Ding, Y. Wei, L. Li, T. Zhang, H. Wang, J. Xue, L.-X. Ding, S. Wang, J. Caro, Y. Gogotsi, MXene molecular sieving membranes for highly efficient gas separation. *Nat. Commun.* **9**, 155 (2018).

71. S. Wan, X. Li, Y. Chen, N. Liu, Y. Du, S. Dou, L. Jiang, Q. Cheng, High-strength scalable MXene films through bridging-induced densification. *Science* **374**, 96–99 (2021).

72. Y. Peng, H. Huang, Y. Zhang, C. Kang, S. Chen, L. Song, D. Liu, C. Zhong, A versatile MOF-based trap for heavy metal ion capture and dispersion. *Nat. Commun.* **9**, 187 (2018).

73. M. Boota, B. Anasori, C. Voigt, M.-Q. Zhao, M. W. Barsoum, Y. Gogotsi, Pseudocapacitive electrodes produced by oxidant-free polymerization of pyrrole between the layers of 2D titanium carbide (MXene). *Adv. Mater.* **28**, 1517–1522 (2016).

74. M. Zhang, K. Guan, Y. Ji, G. Liu, W. Jin, N. Xu, Controllable ion transport by surface-charged graphene oxide membrane. *Nat. Commun.* **10**, 1253 (2019).

75. W. Xin, C. Lin, L. Fu, X.-Y. Kong, L. Yang, Y. Qian, C. Zhu, Q. Zhang, L. Jiang, L. Wen, Nacre-like mechanically robust heterojunction for lithium-ion extraction. *Matter* **4**, 737–754 (2021).

76. C. E. Ren, K. B. Hatzell, M. Alhabeb, Z. Ling, K. A. Mahmoud, Y. Gogotsi, Charge- and size-selective ion sieving through $Ti_3C_2T_x$ MXene membranes. *J. Phys. Chem. Lett.* **6**, 4026–4031 (2015).

77. Y. Shao, L. Wei, X. Wu, C. Jiang, Y. Yao, B. Peng, H. Chen, J. Huangfu, Y. Ying, C. J. Zhang, J. Ping, Room-temperature high-precision printing of flexible wireless electronics based on MXene inks. *Nat. Commun.* **13**, 3223 (2022).

78. C. Zhu, P. Liu, B. Niu, Y. Liu, W. Xin, W. Chen, X.-Y. Kong, Z. Zhang, L. Jiang, L. Wen, Metallic two-dimensional MoS2 composites as high-performance osmotic energy conversion membranes. *J. Am. Chem. Soc.* **143**, 1932–1940 (2021).

79. G. Yang, D. Liu, C. Chen, Y. Qian, Y. Su, S. Qin, L. Zhang, X. Wang, L. Sun, W. Lei, Stable $Ti_3C_2T_x$ MXene-boron nitride membranes with low internal resistance for enhanced salinity gradient energy harvesting. *ACS Nano* **15**, 6594–6603 (2021).

80. L. Ding, D. Xiao, Z. Lu, J. Deng, Y. Wei, J. Caro, H. Wang, Oppositely charged $Ti_3C_2T_x$ MXene membranes with 2D nanofluidic channels for osmotic energy harvesting. *Angew. Chem. Int. Ed. Engl.* **59**, 8720–8726 (2020).

81. J. Chen, W. Xin, X.-Y. Kong, Y. Qian, X. Zhao, W. Chen, Y. Sun, Y. Wu, L. Jiang, L. Wen, Ultrathin and robust silk fibroin membrane for high-performance osmotic energy conversion. *ACS Energy Lett.* **5**, 742–748 (2020).

82. X. Zhu, J. Hao, B. Bao, Y. Zhou, H. Zhang, J. Pang, Z. Jiang, L. Jiang, Unique ion rectification in hypersaline environment: A high-performance and sustainable power generator system. *Sci. Adv.* **4**, eaau1665 (2018).

83. Y. Zhu, K. Zhan, X. Hou, Interface design of nanochannels for energy utilization. *ACS Nano* **12**, 908–911 (2018).

84. S. Zhou, L. Xie, L. Zhang, L. Wen, J. Tang, J. Zeng, T. Liu, D. Peng, M. Yan, B. Qiu, Q. Liang, K. Liang, L. Jiang, B. Kong, Interfacial super-assembly of ordered mesoporous silica–Alumina heterostructure membranes with pH-sensitive properties for osmotic energy harvesting. *ACS Appl. Mater. Interfaces* **13**, 8782–8793 (2021).

85. J. Gao, W. Guo, D. Feng, H. Wang, D. Zhao, L. Jiang, High-performance ionic diode membrane for salinity gradient power generation. *J. Am. Chem. Soc.* **136**, 12265–12272 (2014).

86. H. Cheng, Y. Zhou, Y. Feng, W. Geng, Q. Liu, W. Guo, L. Jiang, Electrokinetic energy conversion in self-assembled 2D nanofluidic channels with janus nanobuilding blocks. *Adv. Mater.* **29**, 1700177 (2017).

87. F. Hashemifar, A. Esfandiar, Oppositely charged MXene fibers as a highly efficient osmotic power generator from sea and river water. *J Mater Chem A* **10**, 24915–24926 (2022).

88. Z. Zhang, L. He, C. Zhu, Y. Qian, L. Wen, L. Jiang, Improved osmotic energy conversion in heterogeneous membrane boosted by three-dimensional hydrogel interface. *Nat. Commun.* **11**, 875 (2020).

89. C. Li, L. Wen, X. Sui, Y. Cheng, L. Gao, L. Jiang, Large-scale, robust mushroom-shaped nanochannel array membrane for ultrahigh osmotic energy conversion. *Sci. Adv.* **7**, eabg2183 (2021).

90. L. Cao, I. C. Chen, C. Chen, D. B. Shinde, X. Liu, Z. Li, Z. Zhou, Y. Zhang, Y. Han, Z. Lai, Giant osmotic energy conversion through vertical-aligned ion-permselective nanochannels in covalent organic framework membranes. *J. Am. Chem. Soc.* **144**, 12400–12409 (2022).
